# Being Present: A single-arm feasibility study of audio-based mindfulness meditation for colorectal cancer patients and caregivers

**DOI:** 10.1371/journal.pone.0199423

**Published:** 2018-07-23

**Authors:** Chloe E. Atreya, Ai Kubo, Hala T. Borno, Blake Rosenthal, Matthew Campanella, John P. Rettger, Galen Joseph, I. Elaine Allen, Alan P. Venook, Andrea Altschuler, Anand Dhruva

**Affiliations:** 1 Department of Medicine and Helen Diller Family Comprehensive Cancer Center (HFCCC), University of California, San Francisco (UCSF), San Francisco, California, United States of America; 2 Kaiser Permanente Division of Research, Oakland, California, United States of America; 3 Department of Psychiatry & Behavioral Sciences, Stanford University School of Medicine, Palo Alto, California, United States of America; 4 Department of Anthropology, History and Social Medicine, UCSF, San Francisco, California, United States of America; 5 Department of Epidemiology and Biostatistics, UCSF, San Francisco, California, United States of America; 6 Osher Center for Integrative Medicine, UCSF, San Francisco, California, United States of America; Vanderbilt University, UNITED STATES

## Abstract

A metastatic cancer diagnosis is associated with high levels of distress in patients and caregivers. Mindfulness interventions can reduce distress and improve quality of life in cancer patients. However, standard mindfulness training relies on in-person instruction, which is often not practical for either patients receiving chemotherapy or their caregivers. In the *Being Present* single arm pilot study, we designed and tested an 8-week audio-based mindfulness meditation program for patients with metastatic colorectal cancer receiving chemotherapy with or without a participating caregiver. The study accrued 33 of 74 (45%) eligible patients consenting together with 20 family caregivers (53 participants total) within nine months. Forty-one participants were evaluable (77%); 10 of 12 cases of attrition were attributable to hospitalization or death. Median participant age was 51 (range 21–78 years); 38% were men. Baseline levels of distress were similar in patients and caregivers. The top reasons for participation cited in pre-intervention interviews were to increase relaxation/calm, improve mood/emotions, and reduce stress/anxiety. In measures of adherence, 59% of responses to weekly texts asking: “Have you practiced today?” were “Yes” and 59% of interviewees reported practicing >50% of the time. Compared to baseline, post-intervention surveys demonstrated significantly reduced distress (p = 0.01) and anxiety (p = 0.03); as well as increased non-reactivity (p<0.01), and feeling at peace (p<0.01). Post-intervention qualitative interviews, where 71% of participants reported benefit, were consistent with quantitative findings. In the interviews, participants spontaneously described reduced stress/anxiety and increased relaxation/calm. Benefits appeared to be accentuated in patient-caregiver pairs as compared to unpaired patients. Seventy-nine percent of participants reported plans for continued practice after study completion. We conclude that the *Being Present* audio-based mindfulness meditation program is of interest to, feasible, and acceptable for patients with metastatic colorectal cancer and caregivers, with initial evidence of efficacy. These results will guide plans for a follow-up study.

**Trial registration**: ClinicalTrials.gov NCT02423720

## Introduction

A cancer diagnosis is associated with high levels of distress, leading to frequent anxiety, depression, fear of recurrence/progression, sleep disturbance, and fatigue in both patients and caregivers [1–8]. The National Comprehensive Cancer Network (NCCN) defines distress as “a multifactorial unpleasant emotional experience of a psychological (i.e. cognitive, behavioral, emotional), social, spiritual and/or physical nature that may interfere with the ability to cope effectively with cancer, its physical symptoms and its treatment” [9]. Screening for psychosocial distress became a Commission on Cancer accreditation requirement in 2015 [10], underscoring the importance of addressing cancer patients’ distress as a part of clinical care [11].

While an estimated 35–45% of cancer patients report psychological distress [1, 12], distress is most common during active treatment [13, 14]. Higher levels of distress and depression are associated not only with worse cancer symptom severity [15] and psychological functioning [16]; but also with inferior survival and prognosis [17]. Additionally, the National Cancer Institute acknowledges the importance of providing supportive care to family members [18]. Family caregivers of cancer patients exhibit high levels of stress and depression, lower subjective well-being, worse physical health, and higher mortality compared to non-caregivers [2–8, 19–25], yet few studies focus on improving outcomes for caregivers [26]. The development of feasible and acceptable evidence-based approaches to address the psychosocial needs of both patients with cancer and caregivers is urgently needed.

To help cope with the distress associated with the diagnosis, disease symptoms, and treatment, individuals affected by cancer are increasingly turning to complementary and integrative medicine [27–30], including mindfulness practices. Mindfulness is defined as moment-to-moment present awareness with an attitude of non-judgment, acceptance, and openness [31]. Mindfulness practice, commonly offered in US medical settings as Mindfulness-Based Stress Reduction (MBSR) [31] or Mindfulness-Based Cognitive Therapy (MBCT) [32], has shown efficacy among cancer patients in reducing psychological distress and improving quality of life [33]. A recent meta-analysis of randomized clinical trials reported that among 15 studies (N = 587 cancer patients), overall distress level was reduced by approximately 40% from baseline following an 8-week mindfulness intervention [34]. However, it is notable that prior studies predominately enrolled breast cancer survivors [35, 36]. The feasibility, acceptability and effectiveness of mindfulness interventions in patients with advanced disease and non-breast cancer diagnoses have been understudied [37–39]. The acceptability of mindfulness-based approaches to men is also not well-established [38]. Existing mindfulness programs (e.g. MBSR) often include over 30 hours of in-person instruction together with 45 minutes of daily home practice, which restricts accessibly to individuals who could derive the greatest benefit: patients with advanced cancer receiving chemotherapy and their busy and burdened caregivers.

To overcome the challenges of physically attending mindfulness classes, recent studies have begun to explore the use of technology to replace face-to-face approaches to deliver mindfulness interventions [40–42]. We previously conducted a pilot study using mindfulness audio CDs for cancer patients undergoing chemotherapy [43] and demonstrated the feasibility and acceptability of a self-paced, non-face-to-face mindfulness intervention and its preliminary efficacy in reducing anxiety. The majority of patients in this pilot study had breast cancer, and 21 of 23 participants (91%) were female.

In order to address these deficiencies in the literature, we focused the *Being Present* study on patients with metastatic colorectal cancer receiving chemotherapy and their caregivers. Although an audio-based mindfulness meditation intervention may be useful to patients with other cancer types, we chose to focus the current study on metastatic colorectal cancer because it is the second leading cause of cancer mortality in both US men and women, accounting for an estimated 50,000 deaths each year. Also, patients with metastatic colorectal cancer and related intestinal malignancies tend to receive similar treatments and live for many months with relatively stable health (median overall survival >2 years) [44], facilitating study completion and potentially derivation of durable benefit from the intervention. Conversely, longer survival comes at a cost for caregivers: the negative effects of caregiving, including a higher mortality rate, are most pronounced in caregivers of patients with advanced cancer [2, 3, 20], particularly when patients have a protracted disease course [25] and are receiving palliative care [4, 21]. A study of colorectal cancer patient-caregiver pairs (N = 212) found that quality of life and depressive symptoms were interdependent in patients and family members [24].

Herein we describe the design and pilot testing of a novel audio-based mindfulness meditation intervention. We conducted focus groups to establish patient and caregiver perceptions of audio-based mindfulness mediation training in order to refine the intervention for further study. We then tested the feasibility, acceptability, and initial evaluations of efficacy of the audio-based mindfulness mediation intervention among patients with colorectal cancer and caregivers. We hypothesized that the patient-caregiver dyad would be central to the success of an intervention without group classes [24, 37, 45]. We postulated that the patient-caregiver relationship might serve as a source of mutual support [46, 47] and a surrogate for community, which is traditionally considered to be an essential ingredient for sustaining mindfulness practices.

## Materials and methods

### Study design and participants

*Being Present* was a two-part study: first, focus groups were conducted as formative research; second, the audio-based mindfulness meditation intervention was piloted. The study protocol was approved by the UCSF Human Research Protection Program Institutional Review Board (IRB # 15–16158) and conducted according to the principles expressed in the Declaration of Helsinki. Participants were recruited by letter or in-person from the UCSF Helen Diller Family Comprehensive Cancer Center (HDFCCC) Gastrointestinal (GI) Oncology Clinic. To limit selection bias, the research coordinator pre-reviewed charts of patients scheduled to be seen in GI Oncology clinic and prompted investigators to discuss the study with all potentially eligible patients. Participants provided written informed consent prior to study procedures.

#### Focus groups

Two focus groups, one for patients and one for caregivers, were conducted simultaneously at the UCSF Osher Center for Integrative Medicine, led by G.J. and A.K. Per protocol, we intended to include 8–10 patients and 8–10 caregivers. Key eligibility criteria included English proficiency and access to a mobile phone and the internet. Patient-specific eligibility requirements were a diagnosis of metastatic colon, rectum, or small bowel adenocarcinoma (intestinal cancer); a life expectancy of ≥6 months; and Eastern Cooperative Group (ECOG) Performance Status ≥2. Participants were asked to complete a demographics and technology use survey. We developed and used a focus group guide (S1 File) to center the two-hour discussions on perceived benefits of and barriers to mindfulness meditation practice and to elicit a range of perspectives/insights from stakeholders for the initial intervention design. The sessions were audio recorded and professionally transcribed verbatim and independently reviewed by three investigators following a general inductive approach for content analysis. Participants were provided with dinner, parking validation, and a $30 gift card.

#### Audio-based mindfulness meditation intervention

The intervention portion of *Being Present* was a single arm study of an 8-week audio-based mindfulness meditation program designed for patients with metastatic intestinal cancer undergoing chemotherapy and their caregivers (S1 Fig). The primary aims of the pilot intervention study were to assess feasibility and acceptability among patients and caregivers; the secondary aim was to conduct preliminary evaluations of efficacy. Content was written under the direction of J.P.R, a clinical psychology-researcher with expertise in the development and implementation of mindfulness interventions (S1 and S2 Tables). In addition to the focus group eligibility criteria, patients were included if they were expected to receive chemotherapy for at least 12 weeks from the time of recruitment; caregivers were only eligible if paired with a participating patient. Subjects with a current meditation practice (>2 sessions or >1 hour total, weekly) or current enrollment in a stress-reduction program were excluded.

Following consent, participants were asked to complete a baseline demographics and technology use survey as well as validated symptom and well-being surveys (see below). A semi-structured pre-intervention interview was conducted by the research coordinator to elucidate reasons for participation, expectations, and prior experience (S1 File). Participants were given a MP3 player (G.G. Martinsen 16 GB) pre-loaded with eight mindfulness meditation tracks as well as a study booklet containing a practice diary. An email was sent each week containing practice instructions, a motivational quote (S4 Table), as well as a link to a discussion of the weekly theme (MP3 file). Participants were instructed to practice 15–20 minutes per day, five days per week, during the 8-week study. They received a text message to their personal cell phone daily at 4 pm (Mosio platform). The majority of text messages contained motivational quotes or practice suggestions. To measure adherence, 13 text messages per participant contained a question to be answered with "Y or N" or a number (S3 Table). At the mid-point and end of the study, additional emails were sent via Research Electronic Data Capture (REDCap) containing links to the same symptom and well-being surveys as were completed at baseline.

Semi-structured post-intervention interviews were conducted by the research coordinator for the qualitative assessment of the effects of study participation and adherence (S1 File). Pre- and post-intervention interviews were audio recorded and professionally transcribed verbatim. Following the post-intervention interview, a list of resources for continued practice was provided via email, comprised of a list of local meditation centers, online guided meditations, CDs/MP3s, mobile apps, and books. A 3-month follow-up REDCap survey was sent to assess for durable impacts of study participation. The date range for participant recruitment was August 2015 –May 2016. Patients were followed for survival outcomes though August 2016.

### Symptom and well-being surveys

The following validated survey instruments were administered at baseline, week 4, and week 8: the NCCN Distress Thermometer [48]; the National Institutes of Health Patient Reported Outcomes Measurement Information System (NIH PROMIS) Anxiety 4a, Depression 4a, Fatigue 6a, Sleep Disturbance 4a, and Global Health Short Forms [49]; the Five Facet Mindfulness Questionnaire Short Form (FFMQ-SF) [50]; and the “Are You at Peace?” one-item spiritual probe [51].

### Analysis

Descriptive statistics were used to summarize demographics, technology use, patient clinical characteristics, and levels/sources of distress. Fisher’s exact tests compared characteristics of evaluable and non-evaluable patients. Paired t-tests compared survey results at baseline to week 4 and week 8 values. The distribution of survey scores was visualized with box plots, histograms, and spaghetti plots using Stata software. Qualitative data analysis of semi-structured interviews followed the framework method [52] and employed Atlas.ti software as outlined in S1 File.

## Results

### Focus groups

Patients and caregivers were invited to participate in the pre-intervention focus groups to gather feedback about audio-based mindfulness meditation training as a means to reduce distress associated with a cancer diagnosis. Invitations were mailed to 34 patients and 25 caregivers (69 total). Six patients and six caregivers participated in the focus groups (18% participation rate). The top reasons for declining participation or late cancelations were illness, scheduling conflicts, and distance/transportation. The participating patients were 67% male (4/6), ages 37–64 years, and half had received chemotherapy in the past month. The participating caregivers were 17% male (1/6), ages 28–68 years (Table 1). The focus groups included both regular meditators and individuals with no meditation experience.

**Table 1:** Demographics and technology use.

	Focus Groups		Intervention			
			Patients		Caregivers	
**Consented Participants (N, %)**	**12**		**33**		**20**	
Gender, male	5	42%	12	36%	8	40%
Age (median, range)^a^	55	28–68	52	23–78	51	21–73
Miles patient lives from UCSF (median, range)	20	3–106	26	2–693		
Caregiver relation to patient						
Significant other	3	50%			13	65%
Parent	0	0%			3	15%
Child	1	17%			4	20%
Friend	2	33%			0	0%
**Demographic Survey Respondents (N, %)**	**11**		**22**		**13**	
Race						
White	7	64%	19	86%	9	69%
Black	1	9%	0	0%	0	0%
Asian	1	9%	1	5%	1	8%
Other	2	18%	2	9%	3	23%
Ethnicity, Latino or Hispanic	3	27%	5	23%	1	8%
Married or long-term partner	10	91%	16	73%	8	62%
Level of education						
College graduate	3	27%	7	32%	2	15%
Professional degree	5	45%	10	45%	10	77%
Currently working, yes	4	36%	8	36%	7	54%
Total annual household income ≥100K	6	55%	13	59%	9	69%
Homeowner, yes	9	82%	16	73%	8	62%
**Technology Use**						
Devices used regularly						
Smartphone	10	91%	19	86%	12	92%
Tablet computer	4	36%	11	50%	5	38%
Laptop computer	5	45%	15	68%	6	46%
Mobile app use, ≥ daily	9	82%	18	82%	13	100%
Text messaging frequency, ≥ daily	9	82%	18	82%	11	85%
Email frequency, ≥ daily	10	91%	19	86%	9	69%

^a^Age data missing from 1 caregiver in a focus group and 7 caregivers in the intervention group.

Two patients who completed baseline surveys were not evaluable.

Both focus groups opened by asking participants to share any prior experience with mindfulness practices. In addition to meditation and yoga, participants cited walking, dancing, and prayer. Mention of these other modalities was incorporated into the intervention text messages and emails. Next, participants were asked what came to mind when they thought of meditation or mindfulness. Here, patients and caregivers provided a wide range of responses. Words associated with meditation included: calm/tranquility/relaxation; health; concentration/focus; consciousness; and blank mode/no thought. A participant who defined meditation as “putting your mind in blank mode” postulated:”I would think mind-*fullness* would be the total opposite.” Another caregiver countered: “for me, meditation is exactly the opposite of blank. It’s to focus in [on] pain or suffering and go to another level and be able to see from outside. Mindfulness, the word that comes to me is empathy.” This discussion led to recognition of the need to provide clear definitions of meditation and mindfulness. Definitions were provided in an audio track introducing the *Being Present* intervention and in the printed study booklet.

Next focus group participants were asked how they thought mindfulness meditation practice could be helpful. Stress relief and relaxation were answers common to both focus groups. A caregiver shared: “it provides a moment of time for yourself… I think it’s a nice little window to have a break and have a calm space to go to.” Patients cited potential utility for management of symptoms including: intrusive thoughts and emotions (e.g. fear), pain, nausea, insomnia, and muscle tension. Based on these responses, *Being Present* includes a Progressive Muscle Relaxation exercise, which is not part of MBSR. Perceived barriers to practice for both patients and caregivers related to having busy lives.

The intervention was then demonstrated, and feedback on the schedule, delivery, and content was requested. There was consensus that an expectation of 20 minutes of practice, 5 days per week without in-person visits was feasible. Both emails and daily text messages were acceptable, with a preference to provide short responses via text. Refinements to the intervention content resulting from the focus groups included the incorporation of quotes from the focus group participants into weekly emails to intervention participants (S4 Table). Additionally, we recorded male and female voice options for every MP3 meditation track based on feedback about voice preferences. We learned that the term “Body Scan,” a foundation of MBSR, triggers anxiety in patients with metastatic cancer due to its association with computed tomography (CT) scans, used to evaluate for tumor progression. As a result, we changed the title of the “Body Scan” track to “Body Awareness Meditation” (S1 Table).

### Audio-based mindfulness meditation intervention

#### Recruitment and retention

**Fig 1:**
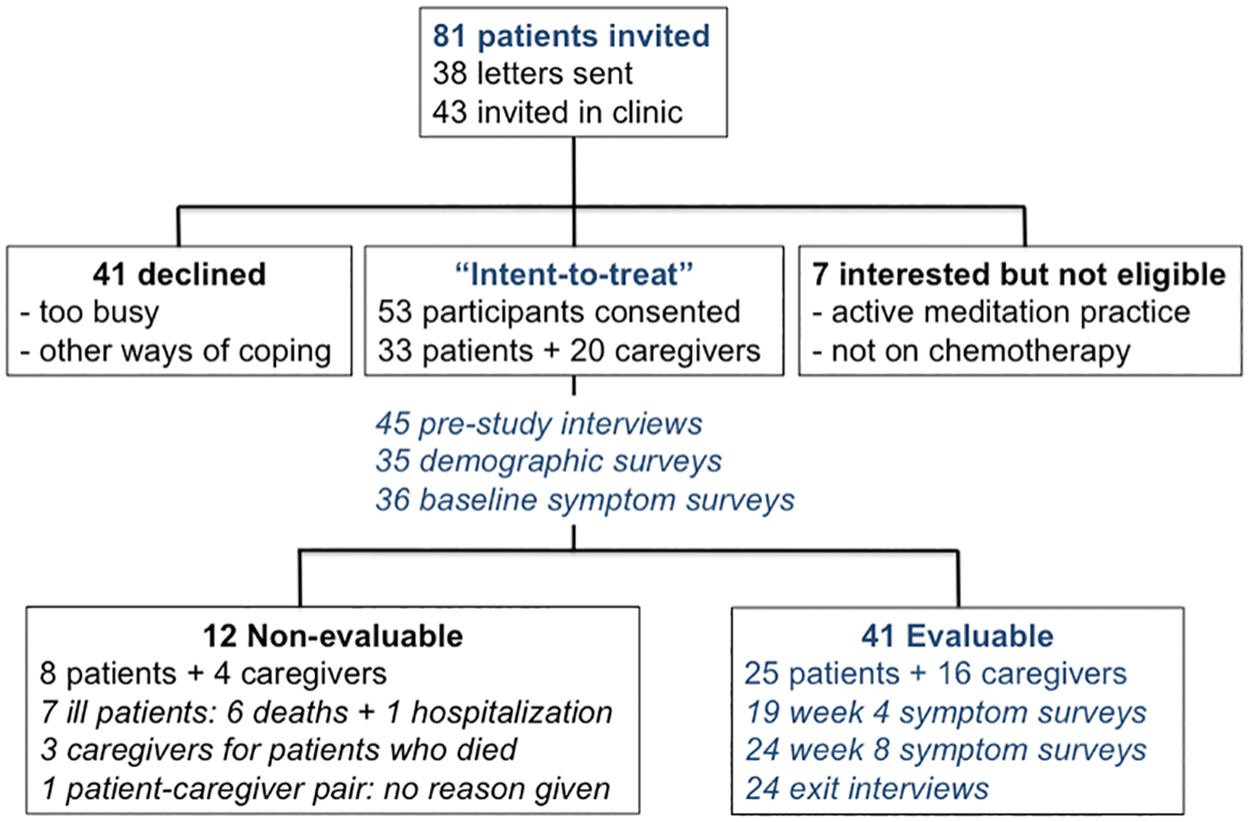
*Being Present* participant flow diagram. Summary of subject recruitment, retention, and data collected. Evaluable was defined by completion of any on-study assessment.

Recruitment occurred over nine months (August 2015-May 2016). Eighty-one patients were invited by letter or in clinic (Fig 1). Reasons given for declining participation included “too much going on” and “other ways of coping” (e.g. yoga, prayer). Seven invited patients were interested but ineligible due to having an active meditation practice (N = 5) or not currently receiving chemotherapy (N = 2). Consent to participate was obtained from 33 of 74 eligible patients (44.6%): 12/33 (36%) consenting patients were recruited by letter; 21/33 (64%) were recruited in clinic. Twenty caregivers of the 33 patients consented to participate (N = 53 total). The median age of consented patients and caregivers was 51 (range 21–78 years); 20/53 (38%) were male. The most common caregiver relationship was significant other (65%) (Table 1).

Eight patients and four caregivers who consented to participate, and in some cases completed baseline assessments, were not evaluable because they completed no on-study assessments (response to text messages, surveys, the study diary, or the exit interview). Compared to evaluable patients, non-evaluable patients had poorer health status, with statistically significantly worse performance status, more hospitalizations and deaths (Table 2). Six non-evaluable patients died unexpectedly early, a median of 2 months (range 0.4–4.4 months) after signing consent; one additional patient was hospitalized soon after consent. Three of the four non-evaluable caregivers were paired with non-evaluable patients who died. Baseline assessments were otherwise similar in the evaluable and non-evaluable groups, and are therefore reported for the intent-to-treat population (Fig 1).

**Table 2:** Patient clinical characteristics.

Clinical Characteristic (N, % or Median, Range)	Intent-to-treat N = 33		Evaluable N = 25		*P*-value^b^
Primary tumor location					
Colon	20	60%	15	60%	
Rectum	7	21%	6	24%	
Appendix or small bowel	4	12%	3	12%	
Anus^a^	1	3%	1	4%	
Years since cancer diagnosis	2	<1–7	1	<1–7	
Line of chemotherapy					
1	11	33%	10	40%	
2	13	39%	9	36%	
>2	9	27%	6	24%	
New chemotherapy on study	10	30%	6	24%	
Chemotherapy at UCSF	18	55%	15	60%	
Baseline ECOG performance status					
0	25	76%	22	88%	0.01
1	6	18%	3	12%	
2	2	6%	0	0%	
Final ECOG performance status					
0	24	73%	22	88%	0.02
1	3	9%	3	12%	
2	1	3%	0	0%	
>2	5	15%	0	0%	
Hospitalization within 12 weeks of consent	5	15%	1	4%	< 0.01
Death, known as of 8/1/2016	7	21%	3	12%	0.01

Eastern Cooperative Group, ECOG; University of California, San Francisco (UCSF).

^a^One patient with metastatic anal cancer enrolled by single patient exception.

^b^Fisher’s exact *P*-values comparing evaluable to non-evaluable patients. All other *P*-values >0.05.

#### Baseline assessments

Demographics of the focus group and intervention participants were comparable, including widespread technology use (Table 1). Both patients and caregivers reported a median baseline distress level in the past week of 5 out of 10 (range 1–8) (Table 3). It is notable that self-reported emotional problems, fears, loss of interest in activities and depression, were roughly twice as common in caregivers compared to patients. As expected, physical problems typically associated with colorectal cancer or chemotherapy (e.g. fatigue, GI symptoms, neuropathy, and change in appearance) were more commonly reported by patients compared to caregivers (Table 3 and S5 Table).

**Table 3:** NCCN Distress Thermometer: Baseline levels and sources of distress.

	Patients (N = 25)		Caregivers (N = 11)	
**Distress Level in past week (median, range)**	5	1–8	5	1–8
**Sources of Distress (N, %)**				
**Emotional Problems**^1^				
Worry	18	72%	10	91%
Nervousness	12	48%	7	64%
Fears	10	40%	9	82%
Sadness	10	40%	7	64%
Loss of interest in activities	6	24%	6	55%
Depression	6	24%	5	45%
**Physical Problems**				
Fatigue	21	84%	5	45%
Nausea	16	64%	0	0%
Sleep	14	56%	6	55%
Eating	14	56%	3	27%
Tingling in hands/feet	13	52%	1	9%
Constipation	13	52%	0	0%
Appearance	12	48%	2	18%
Memory/concentration	8	32%	5	45%
Pain	8	32%	3	27%
Diarrhea	8	32%	0	0%
**Practical Problems**				
Treatment decisions	11	44%	5	45%
Insurance/finances	9	36%	3	27%
**Family Problems**				
Family health issues	8	32%	10	91%
Dealing with partner	6	24%	4	36%
Dealing with children	4	16%	1	9%

National Comprehensive Cancer Network (NCCN) Distress Thermometer screening tool for measuring distress. Selected results at baseline, intent-to-treat population. See S5 Table for complete results.

^1^Distress level is reported on a scale from 0 (no distress) to 10 (extreme distress).

In the pre-intervention semi-structured interviews, prior exposure to meditation ranged from none (N = 17) to having a regular meditation practice in the past (N = 2). Participants shared a wide variety of spontaneously reported reasons for participation or perceived benefits of meditation (Table 4 and S6 Table). Many of these motivations for participation matched sources of distress on baseline surveys. The top reasons for participation given by both patients and caregivers were to foster relaxation/calm, improve mood/emotions or reduce stress/anxiety. As an example, a 43-year-old female patient explained: “I have been struggling over the last year since my diagnosis…The physical is the easy part, it’s the emotional part that’s really difficult, and I really, really need something to help calm my thoughts… Being present, I think is so important, and I haven’t mastered that yet” (S6 Table).

**Table 4:** Analysis of pre-intervention interviews.

	Patients		Caregivers	
N	28		17	
**Prior Meditation Exposure or Related Experience (N, %)**				
No prior related experience	11	39%	6	35%
Meditation	14	50%	7	41%
Yoga	9	32%	7	41%
Prayer/religion	2	7%	2	12%
**Reasons for Participation/Perceived Benefits of Meditation**				
Relaxation/calm—Improve mood/emotions—Reduce stress/anxiety	21	75%	8	47%
Curiosity/interest in meditation	13	46%	7	41%
Focus/train/organize thoughts—avoid racing thoughts	6	21%	1	6%
Meditation has been helpful in the past	5	18%	2	12%
Desire to help research/benefit others	3	11%	4	24%
Caregiver participating to support patient	NA	NA	6	35%
Knows someone who benefits/ has seen meditation work for others	5	18%	0	0%
Discipline/ hope for regular practice	2	7%	3	18%
Help to stay in the present	3	11%	1	6%
Help with sleep	2	7%	2	12%
General health benefits—mind/body/spirit connection	3	11%	1	6%
Lower blood pressure	2	7%	0	0%
Pain management	2	7%	0	0%
Improved communication	0	0%	1	6%
**Preference for MP3 Player vs. Smartphone App or Web-based Program**				
Prefers MP3 player	9	32%	4	24%
Prefers smartphone app or online program	9	32%	6	35%
No preference	10	36%	6	35%

Five patients and two caregivers who completed pre-intervention interviews were not evaluable.

Twelve patient-caregiver pairs were interviewed together.

#### Participant reported outcomes

In post-intervention interviews, 71% of participants reported some form of benefit in response to open-ended questions about effects of study participation (S1 File; Table 5 and S7 Table). The most common spontaneously reported benefit was increased relaxation/calm/sense of peace (33% of interviewees). As the 49-year-old wife of a patient described, “Over time there was definitely a sense of calm and being present that pervaded my mood …I felt everything slow down in a very clear way.” A quarter of the respondents described an attitude adjustment, including cultivation of a more positive mental attitude, or as one 52-year-old male patient said: “it helped me to deal with, or at least contain, the more negative thoughts I was having… It did change my life and helped me through a very difficult time” (S7 Table).

**Table 5:** Analysis of post-intervention interviews.

	Patients		Caregivers	
N	17		7	
**Reported Benefit from Study Participation (N, %)**	12	71%	5	71%
Relaxation/calm/sense of peace	6	35%	2	29%
"Attitude adjustment": (+) mental attitude/ contain (-) thoughts	4	24%	2	29%
Emotional—reduced stress/anxiety	4	24%	1	14%
Improved focus/concentration	2	12%	2	29%
Regular practice /habit forming/ discipline	3	18%	1	14%
Kinder toward self and others	1	6%	1	14%
Physical (unspecified)	1	6%	1	14%
Educational	0	0%	1	14%
**Frequency of Guided Meditation Practice During Study**				
As directed (5x per week for 8 weeks)	3	18%	2	29%
"Most of the time" (>50%)	7	41%	2	29%
~50%	5	29%	2	29%
<50%	2	12%	1	14%
None	0	0%	0	0%
**Barriers to Full Participation**				
Allocating time: busy lives/family obligations	7	41%	5	71%
Struggled with the technology	5	29%	3	43%
Illness/ chemotherapy toxicities	6	35%	0	0%
Travel (specifically difficulty keeping MP3 player charged)	2	12%	1	14%
Preference to do other things	3	18%	0	0%
**Feedback on Study Design or Content**				
Preference for smartphone app/online/no separate device	10	59%	5	71%
Meditation tracks too short	4	24%	2	29%
Meditation tracks too long	0	0%	0	0%
Disliked voice(s) or guided meditation in general	4	24%	2	29%
Liked voice(s)	2	12%	0	0%
Liked text messages	6	35%	3	43%
Disliked text messages	2	12%	0	0%
Difficulty understanding instructions or content	3	18%	2	29%
Increased stress/guilt	4	24%	2	29%
Overly structured	2	12%	1	14%
**Plans for Continued Practice**	14	82%	5	71%
Continued use of *Being Present* audio	4	24%	2	29%
Breathing exercises	5	29%	0	0%
Other guided meditation	1	6%	1	14%
Yoga	2	12%	0	0%
Self-guided meditation	1	6%	0	0%
Intention-setting	1	6%	0	0%
Books	1	6%	0	0%
Involvement of other family members/friends	1	6%	0	0%
Other integration into daily life	0	0%	1	14%

Four patient-caregiver pairs were interviewed together.

**Fig 2:**
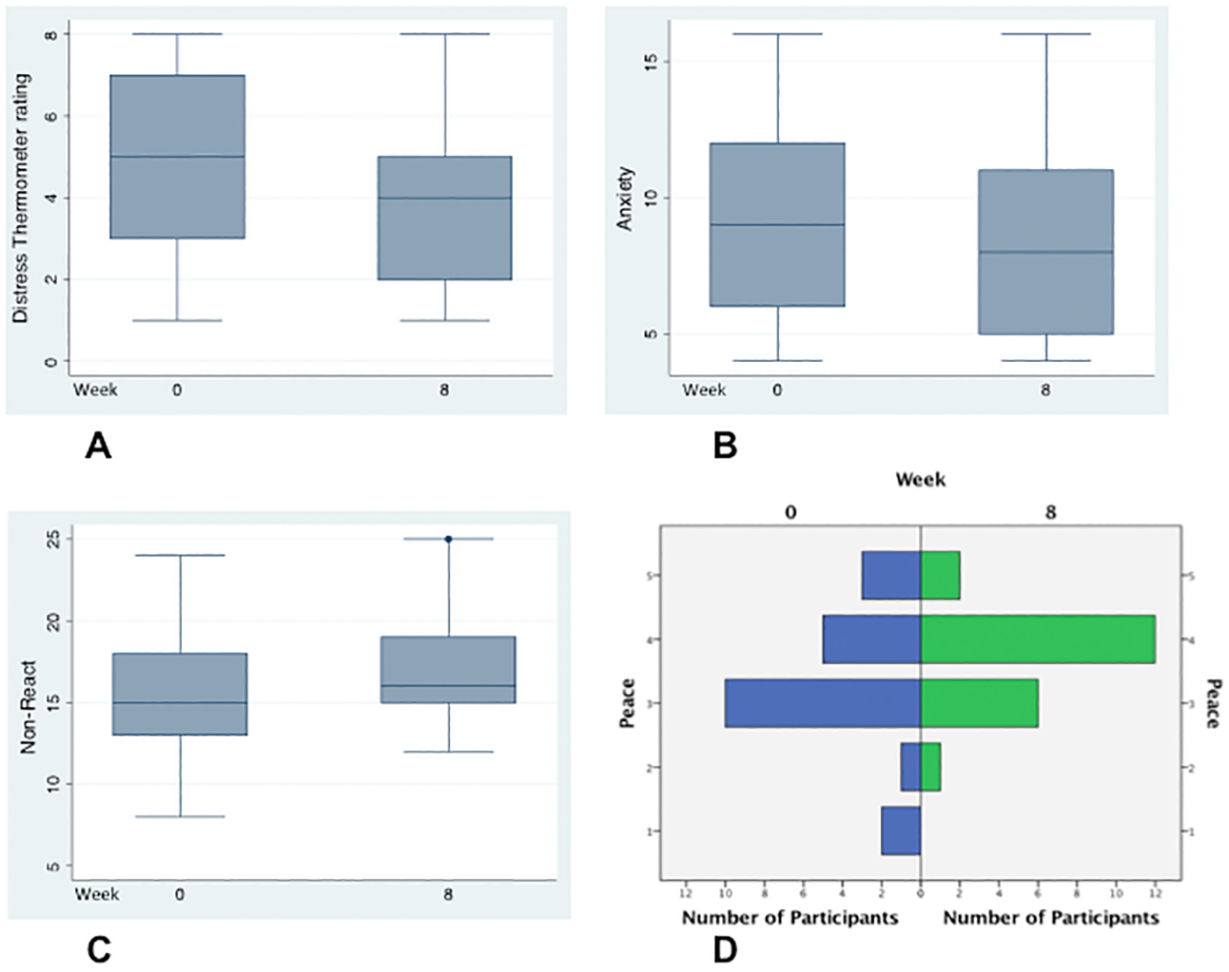
Graphic representations of survey scores at baseline and week 8. N = 24. A) Box plots of National Comprehensive Cancer Network (NCCN) Distress Thermometer ratings: 0 = no distress; 10 = extreme distress. B) Box plots of National Institutes of Health Patient Reported Outcomes Measurement Information System (NIH PROMIS) Anxiety Short Form 4A scores. C) Box plots of Five Facet Mindfulness Questionnaire Short Form (FFMQ-SF) “Non-React” scores. D) Histograms of “Are You at Peace?" one-item spiritual probe ratings: 1 = not at all; 2 = a little bit; 3 = a moderate amount; 4 = quite a bit; 5 = completely. See Table 6 for *P*-values from paired t-tests.

Among participants who completed surveys at baseline and week 8, there was a statistically significant reduction in distress (p = 0.01), anxiety (p = 0.03), and fatigue (p = 0.03); as well as significant improvement in the mindfulness facet, non-reactivity to inner experience (p<0.01); and feeling at peace (p<0.01) (Table 6 and Fig 2). The Cohen’s *d* effect size (*d*) for change in distress was 0.51 (S9 Table). There was a trend toward continued improvement from baseline to week 4, and from week 4 to week 8, with a lower response rate at week 4 (S5 and S8 Tables; S2 Fig). The histograms for “Are you at peace?” shifted up 1 unit from week 0 to week 8, with two participants reporting “not at all” at baseline, whereas all participants reported some degree of feeling at peace by week 8 (Fig 2). No statistically significant changes in depression or global mental health were observed. Regarding the other mindfulness facets, there was a statistically significant worsening in acting with awareness (p = 0.04) and no significant change in describing, non-judging or observing (Table 6; S8 and S9 Tables). Participants with prior exposure to meditation reported greater improvements at week 8 compared to participants with no prior exposure: change in distress, global mental health, non-reacting and feeling at peace all had p<0.01 (S10 Table). The effect size for change in distress was 0.87 in participants with prior meditation exposure (S9 Table).

**Table 6:** Summary of validated survey results.

Measure, mean	All participants (N = 24)			Paired participants^2^ (N = 12)			Unpaired patients (N = 12)		
	Baseline	Week 8	*P*-value	Baseline	Week 8	*P*-value	Baseline	Week 8	*P*-value
**NCCN Distress Thermometer**	4.8	3.8	**0.01**	5.3	3.7	**< 0.01**	4.3	3.9	0.5
**NIH PROMIS** **Short Forms**									
Anxiety 4a	9.6	8.2	**0.03**	10.8	8.5	**0.02**	8.3	7.9	0.6
Depression 4a	7.9	7.1	0.1	7.8	6.4	0.1	8.1	7.8	0.7
Global Mental Health	12.7	13.7	0.1	12.7	14.3	0.2	12.7	13.1	0.6
Fatigue 6a	18.3	15.9	**0.03**	17.4	15.3	0.2	19.4	16.6	0.05
Sleep Disturbance 4a	10.1	11.4	0.06	11	9.1	**< 0.01**	11.8	11	0.5
**FFMQ-SF**									
Acting with Awareness	12.2	10.9	**0.04**	12.6	11.4	0.2	11.7	10.5	0.1
Describing	15.5	16	0.2	15.9	16.7	0.2	15.1	15.4	0.6
Non-judging	13.6	12.5	0.2	12.6	11.5	0.2	14.5	13.4	0.4
Non-reacting	15.3	17.2	**< 0.01**	15.7	18.2	**0.01**	14.9	16.2	0.2
Observing	14.9	15.7	0.2	15.9	16.7	0.3	14	14.7	0.4
**"Are You at Peace?"**^1^	3.3	3.7	**< 0.01**	3	3.5	**< 0.01**	3.6	3.9	0.3

National Comprehensive Cancer Network (NCCN) Distress Thermometer distress screening instrument;

National Institutes of Health Patient Reported Outcomes Measurement Information System (NIH PROMIS);

Five Facet Mindfulness Questionnaire Short Form (FFMQ-SF).

^1^"Are You at Peace?" one-item spiritual probe: 1 = not at all; 2 = a little bit; 3 = a moderate amount; 4 = quite a bit; 5 = completely.

^2^Patient-caregiver pairs. P-values from paired t-tests. P-values <0.05 are in bold. See S5 and S8 Tables for complete results; see S9 Table for effect sizes.

In a pre-specified subset analysis, we did not find notable differences in baseline to week 8 survey results between patients and caregivers (S11 Table). The differences were striking, however, when patient-caregiver pairs were compared to unpaired patients. Benefits were accentuated in paired participants in assessments of distress (p<0.01; *d* = 1.1), anxiety (p = 0.02; *d* = 0.64), non-reacting (p = 0.01; *d* = 0.63), feeling at peace (p<0.01; *d* = 0.68), as well as sleep disturbance (p<0.01; *d* = 0.52) (Table 6 and S9 Table). There were no statistically significant changes in any baseline to week 8 survey results in patients who participated without a caregiver. Unpaired patients did not appear to lack social support—none lived alone and 8/12 (67%) were married versus 5/12 (42%) married members of patient-caregiver pairs.

#### Practice patterns and adherence

In post-intervention interviews, participants reported practicing at a variety of times and in different locations. One 40-year-old female patient explained:

I was able to do it usually in the morning right after everybody left for school…. I was hoping I could fit it in in the evenings after everybody went to bed, but I just found I was too tired then and…it kind of felt like work at that point…I’m able to do it in the acupuncture chair, and then I used it a couple times when I had [a chemotherapy] treatment at the infusion center.

Patient and caregiver pairs reported that they did not practice together. The most common reasons cited for not practicing together related to different schedules and responsibilities. One patient indicated that the MP3 player got in the way of listening to meditation tracks together. Other participants valued taking time for individual self-care. Adherence was comparable in patients and caregivers. Forty-one percent of the interviewees reported practicing >50% of the time (including 7 of the 12 paired participants), with another 29% reporting about 50% adherence to the instruction to practice 5 times per week for 8 weeks (Table 5). Significant improvements in distress (p = 0.02; *d* = 0.77), global mental health (p = 0.03; *d* = 0.71) and feeling at peace (p<0.01; *d* = 0.5) were observed among participants who reported practicing at least “most of the time” (>50%) and who completed both baseline and week 8 surveys (N = 12), whereas changes did not reach statistical significance in survey respondents who reported ≤50% adherence or who did not complete the exit interview (S9 and S12 Tables).

Text messaging was another method used to estimate adherence. There were 36 unique responders out of 41 evaluable participants (89%). We received a median of 6 responses per participant to the 13 questions sent via text (46% response rate). Congruent with the qualitative interview data, 59% of respondents answered “Y” (yes) to the question “Have you practiced today?”, which was asked 9 times over the 8 weeks. Response rates and content were consistent over the course of the study. In week 6, we texted “How many days did you practice mindfulness meditation this week?”–the median answer was 4 (range 2–6) (S13 Table). Questions about adherence sent via text messages provoked stress for some participants. Participants were also asked to fill in a paper practice diary. Despite reminders and provision of a self-addressed stamped envelope, only 8 practice diaries were returned; 2 of which were blank.

The most frequently reported barrier to full participation was allocating time, and this was more common among caregivers vs. patients (71% vs. 41% of interviewees). Thirty-five percent of patients reported that illness or chemotherapy side-effects got in the way of practice. A third of participants cited issues associated with the MP3 player, including track numbering confusion; automatic continuation onto the next track; and battery charging, as hindrances (Table 5 and S7 Table). A 23-year-old female patient offered: “I think to get it on the phone would be key to…regular practice. That’s the one pain point for me personally. I didn’t have [the MP3 player] when I could have relaxed—I was in wait mode” (S7 Table).

Seventy-nine percent of interviewees reported plans for continued practice and 14 participants submitted 3-month follow-up surveys. On the 3-month follow-up survey, 9 of 14 (64%) respondents indicated that participating in the *Being Present* intervention made a lasting impact on their daily life; and 10 of 14 (71%) reported that since the conclusion of the study they had made a conscious effort to practice forms of mindfulness such as meditation and yoga.

#### Feedback on study design and content

Four patients and two caregivers reported negative effects from *Being Present*, specifically stress around not feeling “like I was doing it right,” guilt about not practicing when “you know you should be,” and, in a couple of instances, not understanding the material (Table 5 and S7 Table). Although *Being Present* participants were encouraged to direct questions to the research coordinator, and meditation instructors were available to respond to practice-related inquiries, there were no scheduled check-ins.

Additional feedback included recommendations to build on favorite tracks: breathing exercises and progressive muscle relaxation; to provide longer and shorter track options; and to maintain the male and female voice selections. The design of *Being Present* focused on feasibility for our target population, however the content did not directly address cancer. In post-intervention interviews a few participants did request a track about dealing with cancer and effects of chemotherapy (S7 Table) and we appreciated that one 52-year-old male patient invited “a little more humor because I needed to laugh more during the recovery process.”

## Discussion

To our knowledge, no previous study has examined of the feasibility, acceptability, and effectiveness of a mindfulness meditation program in patients with metastatic intestinal cancer and their family caregivers. The high accrual rate to the *Being Present* intervention demonstrates that an audio-based mindfulness meditation program is of interest to men and women with metastatic intestinal cancers as well as their family caregivers, spanning a wide range of ages. Although a higher percentage of men enrolled compared to most prior mindfulness interventions for integrative cancer care [34], the evaluable study sample included a greater proportion of younger (age <50), female, White and Latino or Hispanic patients relative to the underlying population [44, 53]. UCSF is a referral center and we note that 75% of *Being Present* participants lived outside of San Francisco, some outside of California. Accrual was lower to the in-person focus groups despite broader eligibility, supporting our hypothesis that in-person meetings are challenging for our target population, whereas an audio-based program is feasible and acceptable.

The *Being Present* intervention was designed to be well-matched to the needs and concerns of participants—with input from patient and caregiver focus groups. The observation by focus group participants that the term “Body Scan” triggers anxiety in patients with cancer may be particularly relevant for studies of survivors, who may pursue MBSR programs to mitigate prevalent fear of cancer recurrence [54], typically detected by CT or magnetic resonance imaging body scans. The agreement between issues (baseline sources of distress), expectations (perceived benefits of meditation), and patient reported outcomes following study participation suggests that an audio-based meditation program “that’s manageable and doable, that’s not overwhelming” (S6 Table) and not “too New-agey” (S7 Table), addresses an unmet need for patients, who may live for years with metastatic cancer, and the distressed loved ones who likely will survive them.

Furthermore, we are encouraged by the consistency between the spontaneous expressions of benefit in the post-intervention qualitative interviews and the statistically significant improvements in distress, anxiety, non-reacting and feeling at peace on surveys. Survey results also indicated statistically significant reductions in fatigue (all participants) and sleep disturbance (paired participants). However improved sleep was not mentioned in post-intervention interviews. Of note, the NCCN Distress Thermometer is intended for patients and “Are you at peace” was designed to probe spiritual concerns at end of life. These instruments were not intended for caregivers. Yet we found comparable distress levels in patients and caregivers, and emotional distress in particular may be even higher in caregivers, corroborating previous findings [2–6].

Distress reduction was the main patient-reported outcome assessed in *Being Present*. As patients are living longer with metastatic cancer, more attention should be paid to distress in both patients and caregivers [25]. Distress in cancer patients has been associated with non-adherence to anti-cancer medications; increased number of clinic and emergency room visits, longer hospital stays [55, 56]; worse quality of life and inferior survival [57–59]. In a large pooled analysis, Batty et al found a direct correlation between level of distress and risk of colorectal cancer mortality [57]. Distress in caregivers contributes to distress in patients [24], in addition to negatively impacting the health of caregivers themselves [2–4, 20, 21, 25], and likely other family members (e.g. many of our participants had young children). Thus, we argue that practical and scalable distress reduction strategies, such as audio-based mindfulness meditation programs, that may evade the stigma attached to addressing psychological problems [9], are of value. As with *Being Present*, such programs should be extended to include caregivers as well as patients—as both stakeholders in tailored intervention development and as participants.

Our most intriguing observation may be the suggestion of accentuated benefit in patient-caregiver pairs, compared to unpaired patients. This finding is made more remarkable by the fact that pairs did not practice together or practice more than unpaired patients, and unpaired patients reported good social support. Pairs reported higher baseline levels of distress and anxiety and lower levels of baseline sleep disturbance and non-reactivity to inner experience compared to unpaired patients. It is known that those with greater distress often experience greater benefit from mindfulness and other psychosocial/behavioral interventions, yet all four of the above measures improved significantly in paired participants. We hypothesize that the benefit may relate to what one patient’s wife described:

During the whole process there are, thankfully you could say, few shared experiences. The patient is the one getting the chemo … All those things, and you’re on the outside looking in. I think [the *Being Present* study] could be one of the first opportunities where you’re both going through the same thing… It’s a positive shared experience … It’s a different focus, a healthy focus. (S4 Table)

Two MBSR studies of patient-caregiver pairs provide support for our observations [37, 45]. Birnie et al. found that MBSR improved mood and stress levels in both cancer patients (mostly early stage) and their partners (N = 21 couples). The investigators found that partners’ moods correlated with patients’ stress levels. It was suggested that participating as a couple may improve adherence as well as responses to relationship stress [45]. In a study of patients with advanced stage cancer and their caregivers, Lengacher et al. assessed a modified-MBSR program, including three in-person classes over 6 weeks, in 26 dyads. The investigators observed improved psychological scores in both patients and caregivers, with statistically significant improvements among patients. Over half of screened patients who declined participation cited logistical challenges related to scheduling or transportation [37].

Like any successful pilot, *Being Present* revealed several limitations that can be addressed in a follow-up study. We intended to conduct two focus groups, with 8–10 patients and 8–10 caregivers, however due to challenges associated with scheduling in-person meetings and late cancelations, the focus groups had 6 participants each. A limitation of the intervention was underrepresentation of Black, Asian, and low-income participants. Racial diversity may have been restricted by only providing study materials in English. *Being Present* was a single-arm pilot study, not powered to test efficacy. Participant-reported changes could be the effect of attention. Changes are less likely to be the effect of time in this population, where symptoms typically worsen over the course of treatment and disease progression. More favorable self-reported outcomes among participants with prior meditation exposure may relate to expectancy, which could not be controlled for because a validated expectancy scale was not used. In addition, data on adherence and continued practice was incomplete. We relied on self-reporting and are missing data when participants did not respond to texts, surveys, return the study diary, or complete the post-intervention interview.

Based on what we learned from *Being Present*, we will experiment with replacing in-person focus groups with virtual meetings (e.g. web conferences) with a Patient and Caregiver Advisory Council. Recruitment and diversity may be augmented by expanding the eligibility criteria to include other cancer types; conducting a multi-center trial; translating study materials; and use of social media. A waitlist control, which has been used in studies of cancer survivors [36, 40], would not have been appropriate because of the limited life expectancy of this patient population. Indeed, sample size calculations for future studies will need to take into account that 12 of 53 (23%) consented subjects were not evaluable, primarily due to unforeseen worsening of illness. Developing and testing an appropriate active control group is an important future research direction. A validated expectancy scale will be used and, as discussed below, we will rely on direct measures of adherence and will seek to remove barriers to adherence associated with struggling with the technology. We will also pilot live webinars with a meditation instructor which we hypothesize will increase motivation connected with the feeling of being part of group.

*Being Present* participants offered valuable insights that will guide the design of follow-up studies. One goal of the *Being Present* pilot was to gather information on technology use in our target population. In order to remove possible barriers to participation posed by access to/discomfort with technology, each participant was given an MP3 player. While some participants liked having a separate player for meditation, issues related to the MP3 player predominated. We learned that smartphone use, including mobile applications, was almost universal—irrespective of participant age (Table 1). With *Being Present 2*.*0*, we plan to consolidate to a mobile app or a web-based platform, which will obviate problems with the MP3 player and have the added advantage of automatic data collection about meditation track usage as an objective measure of adherence. Moreover, we believe that difficulties expressed in post-intervention interviews related to practice and understanding content could have been alleviated by talking to a meditation instructor and/or peers, thus highlighting shortcomings of an audio-only program. In the follow-up study we will pilot the feasibility of adding webinars with an instructor, to weigh potential benefits against increased cost and scheduling challenges.

We conclude that the *Being Present* audio-based mindfulness meditation program is feasible, acceptable, and of interest to patients with metastatic intestinal cancers as well as their family caregivers. We have shown preliminary signs of efficacy, with provocative results in patient and caregiver pairs. The effects of mindfulness meditation training in patient-caregiver pairs versus unpaired patients merits further investigation. We anticipate that our findings will be generalizable to patients with other advanced cancer types, who are now living longer than ever, together with the people who care for them.

## Supporting information

S1 Table *Being Present* MP3 tracks. (DOCX)

S2 Table Weekly themes and MP3 track assignments. (DOCX)

S3 Table Text message grid for *Being Present* intervention. (DOCX)

S4 Table Quotes from focus group participants used in intervention emails. (DOCX)

S5 Table NCCN Distress Thermometer screening tool for measuring distress, complete results. (XLSX)

S6 Table Pre-intervention interviews: Quoted reasons for participation. (DOC)

S7 Table Post-intervention interviews: Quotes from participants. (DOC)

S8 Table Complete validated survey results. (XLSX)

S9 Table Effect sizes: Change in validated survey results from baseline to week 8. (DOCX)

S10 Table Summary of validated survey results: Prior meditation exposure. (DOCX)

S11 Table Summary of validated survey results: Patients and caregivers. (DOCX)

S12 Table Summary of validated survey results: >50% vs. ≤50% or unknown adherence. (DOCX)

S13 Table Summary of responses to questions sent in text messages. (DOC)

S1 Fig *Being Present* study logo. Designed by Kameron Allen.
(TIFF)

S2 Fig Spaghetti plots of survey scores at baseline, week 4, and week 8. N = 16. A linear regression line was created from the 3 points for each patient (blue lines) and overall (red dashed line). A) National Comprehensive Cancer Network (NCCN) Distress Thermometer ratings; B) National Institutes of Health Patient Reported Outcomes Measurement Information System (NIH PROMIS) Anxiety Short Form 4A scores; C) Five Facet Mindfulness Questionnaire Short Form (FFMQ-SF) “Non-React” scores; D) “Are You at Peace?" one-item spiritual probe ratings.
(DOC)

S1 File Supplementary methods. (DOCX)

S2 File *Being Present* study protocol. With Institutional Review Board approval notification.
(PDF)

S3 File TREND statement checklist. (PDF)
