# Supplementary material for: Being Present: A single-arm feasibility study of audio-based mindfulness meditation for colorectal cancer patients and caregivers
Source: PLoS One. 2018 Jul 23;13(7):e0199423. doi: 10.1371/journal.pone.0199423 (PMC6056029; doi:10.1371/journal.pone.0199423)
Supplement: S1 Table — (DOCX) [file pone.0199423.s001.docx]

**S1 Table. *Being Present* MP3 Tracks**

| **Track #** | **Track Name** |
| --- | --- |
| 1 | Introduction to Mindfulness |
| 2 | Mindful Breathing Exercise |
| 3 | Progressive Muscle Relaxation |
| 4 | Safe Place Meditation |
| 5 | Attention Awareness Meditation |
| 6 | Body Awareness Meditation |
| 7 | Loving-kindness Meditation |
| 8 | Self-guided Meditation |
