# Supplementary material for: Being Present: A single-arm feasibility study of audio-based mindfulness meditation for colorectal cancer patients and caregivers
Source: PLoS One. 2018 Jul 23;13(7):e0199423. doi: 10.1371/journal.pone.0199423 (PMC6056029; doi:10.1371/journal.pone.0199423)
Supplement: S2 Table — (DOCX) [file pone.0199423.s002.docx]

**S2 Table. Weekly Themes and MP3 Track Assignments**

| **Week #** | **Theme** | **Track(s)** |
| --- | --- | --- |
| 1 | Setting a Healing Intention | 1 & 2 |
| 2 | Working with Beginner's Mind & Patience | 3 |
| 3 | Non-judging | 4 |
| 4 | Working with Practice Challenges & Letting Go | 5 |
| 5 | Trust | 6 |
| 6 | Self-compassion | 7 |
| 7 | Radical Acceptance & Self-reliance | you choose |
| 8 | Extending the Practice Outward | 8 |
