# Supplementary material for: Being Present: A single-arm feasibility study of audio-based mindfulness meditation for colorectal cancer patients and caregivers
Source: PLoS One. 2018 Jul 23;13(7):e0199423. doi: 10.1371/journal.pone.0199423 (PMC6056029; doi:10.1371/journal.pone.0199423)
Supplement: S3 Table — (DOCX) [file pone.0199423.s003.docx]

**S3 Table. Text Message Grid for *Being Present* Intervention**

| **Day** | **Text Message** | **Character Count** |
| --- | --- | --- |
| **WEEK 1: Setting a Healing Intention** | | |
| 1 | (1/4) Welcome to Being Present, a study brought to you by the UCSF Gastrointestinal Oncology Program and the Helen Diller Family Comprehensive Cancer Center. | 157 |
| 1 | (2/4) Each day, you’ll receive a text message. We may ask for a reply. | 71 |
| 1 | (3/4) If you have any questions, call us at: 415-XXX-XXXX or email us at: [xx@ucsf.edu](mailto:xx@ucsf.edu) | 98 |
| 1 | (4/4) If you're having symptoms or an emergency, call your doctor or 911. Please text back ‘Y’ to confirm that you received this text. | 134 |
| 2 | You will be guided in this program through a series of mindfulness practices that will build a strong foundation for your journey. | 130 |
| 3 | Mindfulness is a scientifically validated tool for promoting health. This week develop a plan for when in your day you will set aside time to practice. | 151 |
| 4 | Start your practice by setting a healing intention. Take a moment to think about how you would like your mindfulness practice to come alive in your life. | 153 |
| 5 | Mindfulness practice has shown efficacy in reducing stress and improving quality of life. (Wurtzen 2010) | 104 |
| 6 | The real meditation practice is how we live our lives from moment to moment. (Jon Kabat-Zinn) **Have you practiced today?** Text back Y or N. | 137 |
| 6 | <if Y or y or yes> Wonderful! Keep it up! Feelings come and go like clouds in a windy sky. Conscious breathing is my anchor. ([Thich Nhat Hạnh](http://www.goodreads.com/author/show/9074.Th_ch_Nh_t_H_nh)). | 124 |
| 6 | <if N or n or no> Feelings come and go like clouds in a windy sky. Conscious breathing is my anchor. ([Thich Nhat Hạnh](http://www.goodreads.com/author/show/9074.Th_ch_Nh_t_H_nh)) Take the time to practice meditation today. | 144 |
| 7 | When you get a chance, take a moment to write down your thoughts on your mindfulness meditation practice in the Being Present study booklet. | 141 |
| **WEEK 2: Working with Beginner’s Mind and Patience** | | |
| 8 | Look at everything as though you were seeing it either for the first or last time. Then your time on earth will be filled with glory. (Betty Smith) | 147 |
| 9 | This week focus on observing your thought patterns. Try to let go of the mind’s tendency to be forceful, demanding, and results oriented. | 139 |
| 10 | **Have you had your mindfulness meditation practice today?** Text back Y or N. | 74 |
| 10 | <if Y or y or yes> Great! Hold the intention of practicing with an open mind, the kind of mindset that creates freedom. | 94 |
| 10 | <if N or n or no> Take a moment to practice today. Hold the intention of practicing with an open mind, the kind of mindset that creates freedom. | 127 |
| 11 | The power of mindfulness may rest in its ability to mobilize our natural healing capacities and facilitate a stance of self-kindness, forgiveness, and love. | 156 |
| 12 | We are willing to see everything as if for the first time. We free ourselves of our expectations based on our past experiences. (Jon Kabat-Zinn) | 144 |
| 13 | Good evening! **Have you had your mindfulness practice today?** Text back Y or N. | 77 |
| 13 | <if Y or y or yes> Wonderful! | 13 |
| 13 | <if N or n or no> Try to find just 20 minutes to listen to a track and practice mindfulness meditation. It is worth it! | 101 |
| 14 | Patience is the wisdom to acknowledge that life often unfolds in its own time and to allow it. | 94 |
| **WEEK 3: Non-judging** | | |
| 15 | Intentionally slow down during one of your daily physical activities (e.g. getting dressed) and notice your mental activity and overall sense of your body. | 156 |
| 16 | If you want to conquer the anxiety of life, live in the moment, live in the breath. (Amit Ray) | 94 |
| 17 | To work with the challenges of a wandering or preoccupied mind, we can make great progress by remembering the simplicity of breath awareness. | 141 |
| 18 | Mindfulness is a scientifically validated method for promoting health through the cultivation of specific attitudes toward your self and your world. | 149 |
| 19 | The American Cancer Society recommends meditation as a mind-body process that uses concentration or reflection to relax the body and calm the mind. | 147 |
| 20 | **Did you practice mindfulness today?** Text back Y or N. | 55 |
| 20 | <if Y or y or yes> Good job! If you have time, write down your thoughts about your practice in the Being Present study booklet. | 108 |
| 20 | <if N or n or no> That's okay, life can be busy. Take time for yourself and start your meditation practice for the day. | 102 |
| 21 | Anything we can be doing better in Being Present study? Email us at: [xx@ucsf.edu](mailto:xx@ucsf.edu) | 93 |
| **WEEK 4: Working on Practice Challenges and Letting Go** | | |
| 22 | When you pay attention to boredom it gets unbelievably interesting. (Jon Kabat-Zinn) | 84 |
| 23 | When you need to slow down and come back to yourself, remember you can breathe anywhere. (Thich Nhat Hanh) | 107 |
| 24 | **Do you find the audio meditation tracks easy to follow?** Text back Y or N. | 73 |
| 24 | <if Y or y or yes> Great, we are happy to hear that! | 34 |
| 24 | <if N or n or no> That is good to know. Please contact us with feedback on how to do things better: 415-XXX-XXXX or email us at: [xx@ucsf.edu](mailto:xx@ucsf.edu) | 118 |
| 25 | Observe the space between your thoughts, then observe the observer. (Hamilton Boudreaux) | 88 |
| 26 | Mindfulness meditation has been shown to improve physical and mental health. (Dobos 2015) **Have you practiced today?** Text back Y or N. | 134 |
| 26 | <if Y or y or yes> Keep up the great work! Take time to write down thoughts about your practice in the Being Present study booklet. | 24 |
| 26 | <if N or n or no> No problem, you still have the night ahead of you! Take 15-20 minutes to complete your daily meditation. | 105 |
| 27 | Studies have shown that mindfulness based stress reduction programs enhance psychological well-being immediately and for years after training. (Mitchell 2015) | 159 |
| 28 | Congratulations on completing 4 weeks of the Being Present study! | 66 |
| **WEEK 5: Trust** | | |
| 29 | Your vision will become clear only when you look into your heart. Who looks outside dreams. Who looks inside, awakens. (Carl Jung) | 130 |
| 30 | Good evening! **Have you practiced mindfulness meditation today?** Text back Y or N. | 80 |
| 30 | <if Y or y or yes> Great! Enjoy your practice! | 28 |
| 30 | <if N or n or no> Give mindfulness a chance today. It is worth it. | 48 |
| 31 | Meditation is a way of entering into the quiet that is there buried under the 50,000 thoughts the average person thinks every day. (Deepak Chopra) | 147 |
| 32 | Develop a basic trust in yourself and your feelings. (Jon Kabat-Zinn) | 69 |
| 33 | Between stimulus and response there is a space. In that space is our power to choose our response. In our response lies our growth. (Victor Frankl) | 147 |
| 34 | Mindfulness is cultivated by assuming the stance of an impartial witness to your own experience. (Jon Kabat-Zinn) | 114 |
| 35 | On a scale of 0 to 10, with 0 being not helpful at all and 10 being extremely helpful, **how helpful do you find mindfulness meditation?** | 134 |
| 35 | <If pt texts back <5 or 5> We hope that mindfulness can become more helpful to you in the coming weeks. The key is to keep practicing. | 108 |
| 35 | <If pt texts back 6 or above> We hope that a mindfulness practice can continue to be helpful to you even after this study. | 92 |
| **WEEK 6: Self-compassion** | | |
| 36 | If you want others to be happy, practice compassion. If you want to be happy, practice compassion. (Dalai Lama) | 112 |
| 37 | According to the NIH, regular meditation can reduce chronic pain, anxiety, and high blood pressure. | 99 |
| 38 | Take a moment to write down your thoughts on your mindfulness meditation practice. | 83 |
| 39 | Congratulations - you’ve been practicing mindfulness meditation for 6 weeks! | 77 |
| 40 | We have to look deeply at things in order to see. (Thich Nhat Hanh) | 67 |
| 41 | So what is a good meditator? A good meditator meditates. (Allan Lokos) **Have you done your mindfulness meditation today?** Text back Y or N. | 136 |
| 41 | <if Y or y or yes> Great! | 6 |
| 41 | <if N or n or no> You still have many hours in the day left. Take 20 minutes for you and your practice. | 86 |
| 42 | **How many days did you practice mindfulness meditation this week?** Please respond with a digit (i.e. 1, 2, 3 or 4..) | 114 |
| 42 | <if pt responds 5, 6 or 7> Great! Keep up the good work! Mindfulness meditation is meant to be a daily practice. | 86 |
| 42 | <if pt responds <5> For the best effect, try to take the time to practice mindfulness meditation daily. | 84 |
| **WEEK 7: Radical Acceptance and Self-reliance** | | |
| 43 | The mundane is an opportunity to practice mindfulness. Bring awareness to how water feels as you take sips and the liquid gently moves down your throat. | 152 |
| 44 | Studies have shown that mindfulness meditation reduces stress-related impairments in daily life. (Banks 2015) | 109 |
| 45 | Acceptance means seeing things as they are. Quit trying to force situations to be the way you would like them. This creates tension. (Jon Kabat-Zinn) | 149 |
| 46 | Be happy in the moment, that's enough. Each moment is all we need, not more. ([Mother Teresa](http://www.goodreads.com/author/show/838305.Mother_Teresa)) | 92 |
| 47 | According to the American Cancer Society, cancer patients who meditate have fewer symptoms of stress and mood disturbance than those who do not meditate. | 153 |
| 48 | Happiness is your nature. It is not wrong to desire it. What is wrong is seeking it outside when it is inside. (Ramana Maharshi) | 128 |
| 49 | **Have you already practiced mindfulness meditation today?** Text back Y or N. | 74 |
| 49 | <if Y or y or yes> Great! | 6 |
| 49 | <if N or n or no> There is no time like the present! Refresh your day and mind and practice your mindfulness meditation. | 102 |
| **WEEK 8: Extending the Practice Outward** | | |
| 50 | Meditation is the ultimate mobile device; you can use it anywhere, anytime, unobtrusively. (Sharon Salzberg) | 108 |
| 51 | To be mindful entails examining the path we are traveling, making choices that alleviate suffering & bring happiness to ourselves & around us. (Allan Lokos) | 156 |
| 52 | The people who live in a good community should emanate peace and freshness, the fruits of living in awareness. (Thich Nhat Hanh) | 128 |
| 53 | Use your mindfulness meditation practice to extend your compassion in ever-widening circles, starting with yourself and extending outward. | 139 |
| 54 | Good evening! **Have you practiced mindfulness today?** Text back Y or N. | 69 |
| 54 | <if Y or y or yes> Great! You may also try walking meditation— one step and one breath at a time. | 79 |
| 54 | <if N or n or no> If you are short on time, try walking meditation – one step and one breath at a time. | 85 |
| 55 | We have walked together in mindfulness, learning how to breathe and smile in full awareness, at home, at work, throughout the day. (Thich Nhat Hanh) | 148 |
| 56 | (1/2) Congratulations on completing 8 weeks of the Being Present study! Please take a moment to write down your thoughts in the study booklet. | 143 |
| 56 | (2/2) Find ways to incorporate a mindfulness meditation practice into your daily life. The key is Being Present. Thank you for participating in this study! | 156 |
| 57* | Sorry, if you have any questions or concerns about Being Present, please call us at: 415-XXX-XXXX or email us at: [xx@ucsf.edu](mailto:xx@ucsf.edu) | 138 |

* If participants send a message that deviates from branch logic
