# Supplementary material for: Being Present: A single-arm feasibility study of audio-based mindfulness meditation for colorectal cancer patients and caregivers
Source: PLoS One. 2018 Jul 23;13(7):e0199423. doi: 10.1371/journal.pone.0199423 (PMC6056029; doi:10.1371/journal.pone.0199423)
Supplement: S4 Table — (DOCX) [file pone.0199423.s004.docx]

**S4 Table. Quotes from Focus Group Participants Used in Intervention Emails**

| Mindfulness, as described by one patient with colorectal cancer, is “an experience of peace here and now, awake and aware.” |
| --- |
| Mindfulness allows one to “enjoy the present, and not worry about past and future. That sort of frees you up a little bit.” |
| “I've been using the meditation to really give myself permission to be upset and to be pissed off and to be mad and to be irritable and work through some of that” said one patient with colorectal cancer— “having a way to work through [strong emotions] with the meditation has been helpful for me.” |
| A family member of a patient with colorectal cancer offered, “during the whole process there are, thankfully you could say, few shared experiences. The patient is the one getting the chemo... All those things, and you're on the outside looking in. I think [the *Being Present* study] could be one of the first opportunities where you're both going through the same thing… It's a positive shared experience... It's a different focus, a healthy focus.” |
| “For me it's paying attention to what I’m doing at the present moment. Like if I’m eating, what I’m I eating, how does it taste? What does it feel like?” |
| “Meditation allowed me to just simply stay where I was mentally and to deal.” |
