# Supplementary material for: Being Present: A single-arm feasibility study of audio-based mindfulness meditation for colorectal cancer patients and caregivers
Source: PLoS One. 2018 Jul 23;13(7):e0199423. doi: 10.1371/journal.pone.0199423 (PMC6056029; doi:10.1371/journal.pone.0199423)
Supplement: S6 Table — (DOC) [file pone.0199423.s006.doc]

**S6 Table. Pre-intervention Interviews: Quoted Reasons for Participation**

**Quotes from Patients:**

| I have been struggling over the last year since my diagnosis…The physical is the easy part, it's the emotional part that's really difficult, and I really, really need something to help calm my thoughts… Being present, I think is so important, and I haven't mastered that yet.  *female, age 43* |
| --- |
| I hope it helps me stay in the present because I ... worry, see, and I'm hoping this may help because if you stay right here, you can't really [worry about] what's going to happen a half hour from now, right?  *female, age 78* |
| I'm hopeful that mindfulness will help keep me focused, and keep me calm, and keep me from just going down [with] the craziness of everything that I'm going through right now.  *female, age 50* |
| [I’d like] to go to a place in my head where I can find more of a space to rest.  *female, age 57* |
| I'm interested in learning ways to relax without marijuana and ativan.  *female, age 60* |
| I think it's important to have the right state of mind to get better.  *female, age 41* |
| Honestly, It's been really hard for me to find a program like this that's manageable and doable, that's not overwhelming.  *female, age 43* |

Quotes from Caregivers:

| What I'm hoping is to reduce a little bit of the inner turmoil and stress and emotional ups and downs … that I experience when my daughter has her infusions. My heart just aches for her.  *mother, age 66* |
| --- |
| Stress levels from being a spouse of a cancer patient are pretty high. I‘m hopeful that meditation might be helpful in controlling that stress.  *wife, age 42* |
| Since my mom's been diagnosed with cancer, it's been really hard and a very emotional stage. I am hoping that [*Being Present*] will help me stay mindful of my thoughts but not take off with them and lose track of the moment and what I need to do now.  *daughter, age 21* |
