# Supplementary material for: Being Present: A single-arm feasibility study of audio-based mindfulness meditation for colorectal cancer patients and caregivers
Source: PLoS One. 2018 Jul 23;13(7):e0199423. doi: 10.1371/journal.pone.0199423 (PMC6056029; doi:10.1371/journal.pone.0199423)
Supplement: S7 Table — (DOC) [file pone.0199423.s007.doc]

**S7 Table. Post-intervention Interviews: Quotes from Participants**

**Positive Quotes from Patients (selected)**

| I was able to find a kind of peace I was having trouble finding after all the surgeries and chemo treatments. It helped me to deal with, or at least contain, the more negative thoughts I was having… It did change my life and helped me through a very difficult time.  *male, age 52* |
| --- |
| It expanded my thoughts on meditation and how it can set your attitudes to life a little better or at least controlling aspects of living.  *male, age 52* |
| It's taken me into a whole new path which is something I've been wanting to discover, and this has given me the motivation.  *female, age 62* |
| I definitely felt kinder towards people because I think I felt a little kinder toward myself from some of the meditations.  *female, age 40* |

**Positive Quotes from Caregivers (selected)**

| Over time there was definitely a sense of calm and being present that pervaded my mood...I felt everything slow down in a very clear way.  *female, significant other, age 49* |
| --- |
| I found [the meditations] helpful and they weren't too New-agey or anything like that. I liked that. They were all pretty practical in just focusing you.  *female, significant other, age 46* |
| Overall, it was a great experience and well-organized...easy to do and I had completely positive results… Anything that can create any kind of focus while either going through or taking care of someone going through chemo is a good thing!  *female, significant other, age 49* |
| It was a really good experience because I think it taught me discipline for one. It taught me that, yes indeed, when things are really rough, difficult and emotional, I can still stay true to my agreements and stay true to myself. Stay true to something that I know in my heart can really help me…and help my family.  *mother, age 66* |
| Having committed to the study, I have grown to committing to have a meditation practice! This makes me very happy, as I have never been able to stick with it before. Now I have an experience that has helped me feel less stressful and emotional around my daughter’s illness. Or at least I seem to be able to manage it more gracefully and effectively.  *mother, age 66* |

**S7 Table. Post-intervention Interviews: Quotes from Participants (continuation)**

Constructive Feedback

| It stressed me out more knowing that I wasn't completing the study appropriately… if I missed one day it became infinitely harder for me to keep up the practice. I also never felt like I was doing it right. My mind is always wandering.  *male patient, age 28* |
| --- |
| After a treatment or while recovering from surgery, it was very difficult to breathe as deeply as the meditation was asking, so I felt early on that I was failing.  *male patient, age 52* |
| [Study participation contributed] to “the squeeze”: I know it's ironic, this is supposed to help you, but it's one more thing to do.  *female patient, age 23* |
| It's something that feels really good and really beneficial when you do it, but hard to implement and then that brings up different emotions, like guilt, because you're not doing it and you know you should be.  *female significant other of a patient, age 46* |
| I was very, I don't know if challenged is the right word, but there was a lot of stuff I couldn't wrap my brain around. I found it somewhat contradictory, even.  *female patient, age 72* |
| I think if they can show a video, it'd be more helpful because sometimes they say “do this” [and] I'm not sure…what the instructor meant. If you have an image to show exactly, that would be helpful.  *female patient, age 51* |
| The [text] messages were mostly inspiring and responding was simple. I also thought that the text messages could have added a little more humor because I needed laugh more during the recovery process.  *male patient, age 52* |
| I think to get it on the phone would be key to…regular practice. That's the one pain point for me personally. I didn't have [the MP3 player] when I could have relaxed— I was in wait mode.  *female patient, age 23* |
| It might be nice to have one [track] that specifically talks about cancer, or talks about recovery from treatment. …Maybe on those recovery weeks…I would say, "Okay, I can at least do the 15 minute one about cancer, and chemo being shitty, and feeling awful".  *female patient, age 40* |
