# Supplementary material for: Being Present: A single-arm feasibility study of audio-based mindfulness meditation for colorectal cancer patients and caregivers
Source: PLoS One. 2018 Jul 23;13(7):e0199423. doi: 10.1371/journal.pone.0199423 (PMC6056029; doi:10.1371/journal.pone.0199423)
Supplement: S9 Table — (DOCX) [file pone.0199423.s009.docx]

**S9 Table. Effect Sizes: Change in Validated Survey Results from Baseline to Week 8**

|  | **All** | | **Pair (+)** | **Pair (–)** | **Patients** | **Care-givers** | **Meditate(+)** | **Meditate(–)** | **Adhere (+)** | **Adhere (–)** |  |
| --- | --- | --- | --- | --- | --- | --- | --- | --- | --- | --- | --- |
| **NCCN Distress Thermometer** | **0.51** | | **1.1** | 0.17 | 0.44 | **0.81** | **0.87** | 0.13 | **0.77** | 0.27 |  |
| **NIH PROMIS**  **Short Forms** |  | |  |  |  |  |  |  |  |  |  |
| Anxiety 4a | 0.36 | | **0.64** | 0.10 | 0.20 | **0.99** | 0.38 | 0.43 | 0.34 | 0.36 |  |
| Depression 4a | 0.24 | | 0.41 | 0.07 | 0.06 | **1.2** | **0.50** | 0.06 | 0.21 | 0.25 |  |
| Global Mental Health | 0.31 | | **0.51** | 0.11 | 0.18 | **0.89** | **0.69** | 0.27 | **0.71** | 0.27 |  |
| Fatigue 6a | 0.39 | | 0.35 | 0.42 | 0.29 | **0.67** | 0.45 | **0.52** | 0.49 | 0.30 |  |
| Sleep Disturbance 4a | 0.34 | | **0.52** | 0.19 | 0.26 | **0.77** | 0.43 | 0.17 | **0.63** | 0.19 |  |
| **FFMQ-SF** |  | |  |  |  |  |  |  |  |  |  |
| Acting with Awareness | 0.30 | | 0.27 | 0.31 | 0.15 | **0.92** | 0.30 | 0.23 | 0.24 | 0.40 |  |
| Describing | 0.24 | | 0.34 | 0.13 | 0.25 | 0.19 | 0.35 | 0.14 | 0.15 | 0.44 |  |
| Non-judging | 0.25 | | 0.23 | 0.27 | 0.20 | 0.43 | **0.62** | 0.13 | 0.21 | 0.29 |  |
| Non-reacting | 0.47 | | **0.63** | 0.31 | 0.45 | **0.59** | **0.59** | 0.29 | 0.44 | 0.49 |  |
| Observing | 0.22 | | 0.25 | 0.20 | 0.16 | 0.47 | 010 | 0.36 | 0.21 | 0.25 |  |
| **"Are You at Peace?"** | 0.46 | | **0.68** | 0.29 | 0.36 | **1.3** | **0.66** | 0.16 | **0.50** | 0.39 |  |
|  | |  |  |  |  |  |  |  |  |  |  |
| National Comprehensive Cancer Network (NCCN) Distress Thermometer distress screening instrument; | | | | | | | | | | | |
| National Institutes of Health Patient Reported Outcomes Measurement Information System (NIH PROMIS);  Five Facet Mindfulness Questionnaire Short Form (FFMQ-SF). "Are You at Peace?" one-item spiritual probe. | | | | | | | | | | | |
| All = all participants; Pair(+) = paired participants; Pair(–) = unpaired participants, see Table 6. Patients and Caregivers, see Table S11. Meditate(+) = prior meditation exposure; Meditate(–) = no prior meditation exposure, see Table S10. Adhere(+) = >50% adherence; Adhere(–) = ≤50% or unknown adherence, see Table S12. Cohen’s *d* effect sizes ≥0.5 are in bold. | | | | | | | | | | | |
