# Supplementary material for: Being Present: A single-arm feasibility study of audio-based mindfulness meditation for colorectal cancer patients and caregivers
Source: PLoS One. 2018 Jul 23;13(7):e0199423. doi: 10.1371/journal.pone.0199423 (PMC6056029; doi:10.1371/journal.pone.0199423)
Supplement: S10 Table — (DOCX) [file pone.0199423.s010.docx]

**S10 Table. Summary of Validated Survey Results: Prior Meditation Exposure**

|  | **All participants**  **(N=24)** | | | | **Prior meditation exposure**  **(N=12)**^3^ | | | **No meditation exposure**  **(N=9)**^2^ | | |  |
| --- | --- | --- | --- | --- | --- | --- | --- | --- | --- | --- | --- |
| Measure, mean | Baseline | | Week 8 | *P*-value | Baseline | Week 8 | *P*-value | Baseline | Week 8 | *P*-value |  |
| **NCCN Distress Thermometer** | 4.8 | | 3.8 | **0.01** | 5.2 | 3.4 | **< 0.01** | 4.4 | 4.1 | 0.73 |  |
| **NIH PROMIS**  **Short Forms** |  | |  |  |  |  |  |  |  |  |  |
| Anxiety 4a | 9.6 | | 8.2 | **0.03** | 10.6 | 9.2 | 0.09 | 8.3 | 6.8 | 0.2 |  |
| Depression 4a | 7.9 | | 7.1 | 0.1 | 8.7 | 7.1 | 0.08 | 7.3 | 7.1 | 0.7 |  |
| Global Mental Health | 12.7 | | 13.7 | 0.1 | 12.1 | 14.5 | **< 0.01** | 13.8 | 13 | 0.4 |  |
| Fatigue 6a | 18.3 | | 15.9 | **0.03** | 18.7 | 15.9 | 0.1 | 19 | 15.4 | **0.02** |  |
| Sleep Disturbance 4a | 10.1 | | 11.4 | 0.06 | 11.6 | 10 | 0.08 | 10.3 | 9.7 | 0.6 |  |
| **FFMQ-SF** |  | |  |  |  |  |  |  |  |  |  |
| Acting with Awareness | 12.2 | | 10.9 | **0.04** | 13.2 | 11.8 | 0.2 | 10.6 | 9.7 | 0.2 |  |
| Describing | 15.5 | | 16 | 0.2 | 16.1 | 16.8 | 0.2 | 14.8 | 15.1 | 0.6 |  |
| Non-judging | 13.6 | | 12.5 | 0.2 | 14.5 | 12 | 0.05 | 12.3 | 13 | 0.5 |  |
| Non-reacting | 15.3 | | 17.2 | **< 0.01** | 14.8 | 17.3 | **< 0.01** | 16.1 | 17.3 | 0.4 |  |
| Observing | 14.9 | | 15.7 | 0.2 | 16.1 | 16.4 | 0.7 | 13.9 | 15.2 | 0.2 |  |
| **"Are You at Peace?"**^1^ | 3.3 | | 3.7 | **< 0.01** | 3 | 3.7 | **< 0.01** | 3.8 | 3.9 | 0.6 |  |
|  | |  |  |  |  |  |  |  |  |  |  |
| Note that current meditation practice (>2 sessions or >1 hour total, weekly) was an exclusion criteria.  Two of 10 respondents with prior exposure reported having a regular meditation practice in the past.  National Comprehensive Cancer Network (NCCN) Distress Thermometer distress screening instrument; | | | | | | | | | | | |
| National Institutes of Health Patient Reported Outcomes Measurement Information System (NIH PROMIS);  Five Facet Mindfulness Questionnaire Short Form (FFMQ-SF). | | | | | | | | | | | |
| ^1^"Are You at Peace?" one-item spiritual probe: 1=not at all; 2=a little bit; 3=a moderate amount; 4= quite a bit;  5=completely. ^2^N=10 for Distress; N=8 for Global Mental Health and Peace. ^3^N=11 for Anxiety, Depression, and Sleep Disturbance. *P*-values from paired t-tests. *P*-values <0.05 are in bold. | | | | | | | | | | | |
