# Supplementary material for: Being Present: A single-arm feasibility study of audio-based mindfulness meditation for colorectal cancer patients and caregivers
Source: PLoS One. 2018 Jul 23;13(7):e0199423. doi: 10.1371/journal.pone.0199423 (PMC6056029; doi:10.1371/journal.pone.0199423)
Supplement: S11 Table — (DOCX) [file pone.0199423.s011.docx]

**S11 Table. Summary of Validated Survey Results: Patients and Caregivers**

|  | **All participants**  **(N=24)** | | | | **Patients**  **(N=17)**^2^ | | | **Caregivers**  **(N=5)**^3^ | | |  |
| --- | --- | --- | --- | --- | --- | --- | --- | --- | --- | --- | --- |
| Measure, mean | Baseline | | Week 8 | *P*-value | Baseline | Week 8 | *P*-value | Baseline | Week 8 | *P*-value |  |
| **NCCN Distress Thermometer** | 4.8 | | 3.8 | **0.01** | 4.8 | 3.8 | 0.06 | 5 | 3.8 | 0.08 |  |
| **NIH PROMIS**  **Short Forms** |  | |  |  |  |  |  |  |  |  |  |
| Anxiety 4a | 9.6 | | 8.2 | **0.03** | 9.2 | 8.4 | 0.2 | 10.8 | 7.6 | 0.08 |  |
| Depression 4a | 7.9 | | 7.1 | 0.1 | 8 | 7.8 | 0.7 | 7.8 | 5 | **< 0.01** |  |
| Global Mental Health | 12.7 | | 13.7 | 0.1 | 12.7 | 13.3 | 0.3 | 12.8 | 15 | 0.3 |  |
| Fatigue 6a | 18.3 | | 15.9 | **0.03** | 18.6 | 16.8 | 0.1 | 17.5 | 13.3 | 0.1 |  |
| Sleep Disturbance 4a | 10.1 | | 11.4 | 0.06 | 11.7 | 10.6 | 0.2 | 10.6 | 8.6 | 0.08 |  |
| **FFMQ-SF** |  | |  |  |  |  |  |  |  |  |  |
| Acting with Awareness | 12.2 | | 10.9 | **0.04** | 11.6 | 11 | 0.3 | 14 | 10.8 | **0.04** |  |
| Describing | 15.5 | | 16 | 0.2 | 15.5 | 16 | 0.2 | 15.6 | 16 | 0.7 |  |
| Non-judging | 13.6 | | 12.5 | 0.2 | 14 | 13.1 | 0.4 | 12.2 | 10.4 | 0.3 |  |
| Non-reacting | 15.3 | | 17.2 | **< 0.01** | 14.8 | 16.6 | **0.02** | 17.2 | 19.2 | 0.2 |  |
| Observing | 14.9 | | 15.7 | 0.2 | 14.7 | 15.3 | 0.4 | 15.8 | 17.2 | 0.3 |  |
| **"Are You at Peace?"**^1^ | 3.3 | | 3.7 | **< 0.01** | 3.3 | 3.7 | 0.05 | 3.2 | 3.8 | 0.07 |  |
|  | |  |  |  |  |  |  |  |  |  |  |
| National Comprehensive Cancer Network (NCCN) Distress Thermometer distress screening instrument; | | | | | | | | | | | |
| National Institutes of Health Patient Reported Outcomes Measurement Information System (NIH PROMIS);  Five Facet Mindfulness Questionnaire Short Form (FFMQ-SF). | | | | | | | | | | | |
| ^1^"Are You at Peace?" one-item spiritual probe: 1=not at all; 2=a little bit; 3=a moderate amount; 4= quite a bit;  5=completely. ^2^N=18 for Distress; N=16 for Peace, Global Mental Health and Sleep Disturbance. ^3^N=6 for Distress and Fatigue. *P*-values from paired t-tests. *P*-values <0.05 are in bold. | | | | | | | | | | | |
