# Supplementary material for: Being Present: A single-arm feasibility study of audio-based mindfulness meditation for colorectal cancer patients and caregivers
Source: PLoS One. 2018 Jul 23;13(7):e0199423. doi: 10.1371/journal.pone.0199423 (PMC6056029; doi:10.1371/journal.pone.0199423)
Supplement: S13 Table — (DOC) [file pone.0199423.s013.doc]

**S13 Table. Summary of Responses to Questions Sent in Text Messages**

|  |  |  |  |  |
| --- | --- | --- | --- | --- |
| **Text Questions** | **Text Responses** | | | |
|  | Yes, (N, %) | | No, (N, %) | |
| Have you practiced today? (asked 9 times over 8 weeks) | 84 | 59% | 58 | 41% |
| Do you find the audio meditation tracks easy to follow? | 12 | 86% | 2 | 14% |
|  | N | median, range | | |
| On a scale of 0 to 10, how helpful do you find mindfulness meditation?^1^ | 15 | 8 2-10 | | |
| How many days did you practice mindfulness meditation this week? | 13 | 4 2-6 | | |
| ^1^Asked at the end of week 6. See S4 Table for complete text message grid. |  |  |  |  |
|  |  |  |  |  |
|  |  |  |  |  |
|  |  |  |  |  |
|  |  |  |  |  |
