# Supplementary figures and images for: Being Present: A single-arm feasibility study of audio-based mindfulness meditation for colorectal cancer patients and caregivers
Source: PLoS One. 2018 Jul 23;13(7):e0199423. doi: 10.1371/journal.pone.0199423 (PMC6056029; doi:10.1371/journal.pone.0199423)

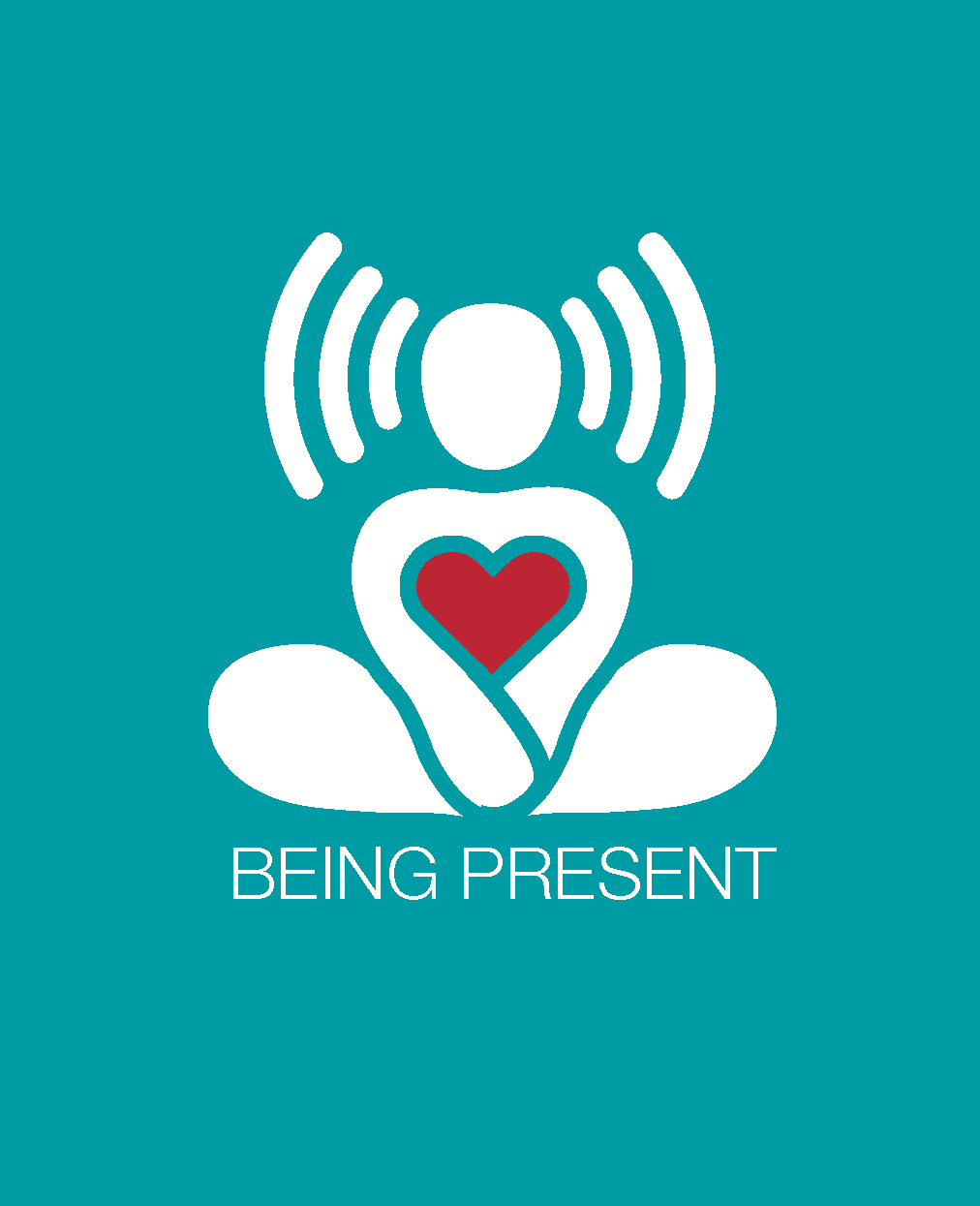

Supplement: S1 Fig — Designed by Kameron Allen. (TIFF) [file pone.0199423.s014.tiff]
