# Supplementary material for: Being Present: A single-arm feasibility study of audio-based mindfulness meditation for colorectal cancer patients and caregivers
Source: PLoS One. 2018 Jul 23;13(7):e0199423. doi: 10.1371/journal.pone.0199423 (PMC6056029; doi:10.1371/journal.pone.0199423)
Supplement: S2 Fig — N = 16. A linear regression line was created from the 3 points for each patient (blue lines) and overall (red dashed line). A) National Comprehensive Cancer Network (NCCN) Distress Thermometer ratings; B) National Institutes of Health Patient Reported Outcomes Measurement Information System (NIH PROMIS) Anxiety Short Form 4A scores; C) Five Facet Mindfulness Questionnaire Short Form (FFMQ-SF) “Non-React” scores; D) “Are You at Peace?" one-item spiritual probe ratings. (DOC) [file pone.0199423.s015.doc]

**A B**

**C D**
