# Supplementary material for: Being Present: A single-arm feasibility study of audio-based mindfulness meditation for colorectal cancer patients and caregivers
Source: PLoS One. 2018 Jul 23;13(7):e0199423. doi: 10.1371/journal.pone.0199423 (PMC6056029; doi:10.1371/journal.pone.0199423)
Supplement: S1 File — (DOCX) [file pone.0199423.s016.docx]

**S1 File. Supplementary Methods**

**Power and Sample Size Calculation**

The power calculation for intervention feasibility (the primary objective) was based on the proportion of participants who successfully completed the intervention, defined as ≥70% of participants listening to the meditation recordings and completing a follow-up assessment (i.e. ≥70% evaluable participants). Using a 1-sided 1-sample binomial test with α = 0.05, 20 patients or caregivers, and an expected successful completion proportion of 70%, we would have 80% power to reject the null hypothesis if 48% or fewer participants successfully completed the protocol. In other words, the intervention would be considered *not* feasible for patients if fewer than 10 patients completed the protocol, and *not* feasible for caregivers if fewer than 10 caregivers completed the protocol. Because patients could participate without a caregiver, enrollment continued until a total of 20 caregivers had consented.

**Focus Group Guide: Being Present Mindfulness Meditation Study**

**I. INTRODUCTIONS**

Welcome. Thank you very much for coming. I am __ (Facilitator)___. I will be the facilitator of this discussion group. This is _________ another other researchers on the study who will be helping to facilitate or taking notes.

**II. PURPOSE**

The purpose of this discussion is to gather feedback about audio-based mindfulness meditation training as a means to reduce distress associated with a cancer diagnosis among individuals treated with chemotherapy for intestinal cancer, and family members/friends these individuals.

**III. PROCEDURES**

A. Opinions:

We are interested in all of your ideas, comments, and suggestions. There are no right and wrong answers. All comments--both positive and negative--are needed. I’d like to set some ground rules.

First, please feel free to disagree with one another, but please don’t criticize other people’s opinions.

Second, please speak one at a time so that we can hear each one of you when you speak.

Third, we would like you all to participate, and so I may call on you from time to time.

Fourth, to preserve confidentiality for everyone in the group, please only use first names, and do not tell anyone outside the group what any particular person in the group said.

B. Confidentiality:

We will audio record the discussion so that we can accurately capture all of your ideas without having to take detailed notes. All the information you provide will be kept confidential and will only be used by the people directly involved with this project. Your real names will not be used in any reports or publications about this research. If you prefer not to be tape recorded, you are welcome to leave the group at any time. Also, you can choose not to address any topic raised during the discussion.

C. Housekeeping:

Our discussion will last approximately two hours. Please turn off your cellular phones, pagers, or any electronic device that emits noise so that they will not disturb our discussion.

The bathroom is ___[location]____. Please feel free to go to bathroom or have refreshments whenever you like. Most importantly, we want you to feel very relaxed during this meeting. If you have any other need that we haven’t mentioned, don’t hesitate to let us know.

**D. Consent**

Now I will talk about the consent form. [Facilitator will highlight key points.] Please take your time to read it over, and I can answer any question you have. [Pause for reading form.]

What questions do you have?

Collect consent and **Demographic Forms**

May I have your permission to begin?

**I. Warm up**

1. To begin, let’s go around the room and get acquainted. Tell us your first name, and tell us what if any experiences you’ve had with meditation, yoga, or other mindfulness practices.

**II. Knowledge and definitions**

1. Take a few minutes to think about and write down the answers to these questions, and then we’ll go around the room and talk about them

- When you think of meditation, what comes to mind?
- When you think of mindfulness, what comes to mind?

**III. Benefits/barriers**

We are specifically interested in helping patients and family members/friends of patients who are receiving chemotherapy. If you or your loved one is not currently receiving chemotherapy treatment, please think back to that time when answering these next few questions.

1. What, if anything, makes you interested in mindfulness practices like meditation and yoga while undergoing chemo?
   1. Probe: why are you interested?
   2. Probe: Tell me more about that.
2. How do you think a regular mindfulness practice could help you?
   1. Probe: In what other ways could it help you?
   2. Probe: Tell me more about that.
3. What do you think might get in the way of developing a practice?
   1. Probe: Anything else that might get in the way?

**IV. Intervention Demonstration**

Now, please let us describe the study we will be conducting. We would like your feedback regarding this study. The goal of this research study is to test whether we can incorporate stress-reduction techniques such as meditation into the standard care for cancer patients and their friends/families. We are also interested in finding out what effect such a program can have on the stress level of cancer patients and their families. The study involves answering questions three times during the 8-week study period, once at the clinic, and the rest can be done at the clinic or at home at your convenience. Participants will receive several meditation exercises as mp3 files in their smartphone, tablet or mp3 player. They will be asked to practice meditation for about 20minutes a day, at least 5 days a week for 8 weeks. There will be a booklet and instruction for them to follow for the 8-week period. Referencing the draft intervention consent form, explain additional study details: audio, texts and email; no in person meetings, goal to recruit patient-family/friend pairs etc. All participants will be patients receiving chemotherapy for intestinal cancers and family members or friends of these patients.

We are now going to play a sample of the audio for you so you can get a feel for it. Then we’ll ask you some questions about it.

1. So first let’s listen to a two samples of the audio *(play the first audio clip, and ask a and b; then play second audio clip and ask a and b)*
   1. How do you feel about the sound of the voice? (for each audio clip)
   2. Is this a voice you would want to listen to on a daily basis for several weeks if he/she were provided guided meditation/ instructions for a meditation program?
2. Afte*r playing both the male and female voices, ask:*
   1. Would you prefer to listen to a single voice for all of the guided meditation exercises, or different voices for different tracks?
   2. Would you like to have a choice between male vs. female voice for all tracks?
3. One of the meditation exercises is a traditional mindfulness exercise called the “body scan”. If I say “body scan” what does that bring up for you?
   1. Probe: What comes to mind?
   2. Probe: Tell me more about that…
4. Describe program schedule and solicit feedback. The research study would have the participants practice mindfulness exercises using the recordings for 8 weeks, 20 minutes a day, 5 days a week.
   1. How does this schedule sound to you?
   2. How do you think it would fit into your daily life?
   3. What kinds of changes would you have to make, and how easy or hard would those be for you?
5. As part of the program, participants would receive texts periodically with practice reminders, quotes, tips or questions as well as weekly emails with practice instructions, but there would be no in-person meetings with the researchers/instructor. Texts would be sent daily with reminders to meditate, and short quotes [provide example]. Emails would be sent 1-2x per week with more information, for example providing the theme for the week…
   1. How would you feel about receiving daily texts that remind you to meditate?
      1. Probe: would they be helpful? Why or why not?
      2. Probe: would they be annoying? Why or why not?
   2. what about texts asking for a brief response (Y,N or a #)?
      1. what about texts with inspirational quotes or poetry?
   3. How would you feel about receiving emails a couple of times a week?
      1. Probe: Do you think you would read them? Why or why not?
6. How do you feel when you think about getting the recorded exercises on an MP3 player and follow up texts and emails, with no in-person meetings?
   1. Does it make you more/less inclined to participate? Tell me about that.
   2. Would you prefer this mp3 method or an online format, where you go on a website, and listen to the meditation at home or download onto any device and listen?
   3. Would you like to have interaction with a teacher or other participants? Why or why not?
   4. Would you like to have interaction with other participants? For example, through an online forum or conversation board on a website? You can think of it as a private group on social media only for the participants of this particular study where you can post questions or share experiences.
      1. Why or why not?

The program is designed for patients and family member/friend pairs. What do you think about that?

- 1. What do you think the benefits might be to offering it to pairs?
  2. What might be the drawbacks?

1. Any other thoughts about this meditation program?
   Anything we didn’t ask you about?

**Focus Groups Consolidated Criteria for Reporting on Qualitative Studies (COREQ)**^1^

**Domain 1: Research team and reflexivity**

***Personal Characteristics*:** Dr. Galen Joseph, PhD, Associate Professor of Anthropology, History, and Social Medicine at UCSF and Dr. Ai Kubo, MPH, PhD a research scientist and cancer epidemiologist at the Kaiser Permanente Northern California Division of Research facilitated the focus groups.

***Relationship with participants***: G.J. and A.K. did not have any prior relationship with the participants. Focus group participants did not have prior knowledge of who the facilitators would be. Investigators who did have an established relationship with focus group participants (C.E.A. and A.P.V.) were excluded to avoid bias in responses.

**Domain 2: Study design**

***Theoretical framework****:* the methodological orientation was a general inductive approach using content analysis.

***Participant selection***:
- Sampling: by convenience and consecutive, based on availability to attend the focus group sessions.

- Method of approach: by mail and face-to-face.

- Sample size: 6 patients and 6 caregivers participated in the focus groups

- Non-participation: invitations were mailed to 34 patients and 25 caregivers (69 total). The top reasons for declining participation or late cancelations were illness, scheduling conflicts, and distance/transportation.
***Setting:***

- Setting of data collection: focus groups were conducted simultaneously at the UCSF Osher Center for Integrative Medicine on May 12, 2015. Dinner and parking validation were provided.

- Presence of non-participants: besides the facilitators and participants, H.T.B. (medical resident) and B.R. (research coordinator) were present to help facilitate and take notes.

- Description of sample: Patients were ages 37-64 years; 4 of 6 were male, all had a diagnosis of metastatic colorectal cancer and half had received chemotherapy in the past month. Caregivers were ages 28-69 years; 1 of 6 was male.
***Data collection:***

- A focus group guide was created; the same guide was used by both facilitators and is provided in S1 File (above).

- Repeat interviews were not conducted.

- Audio recordings were used to collect the data.

- Field notes were made during/after the focus groups by H.T.B. and B.R.
- Duration: The focus group duration was two hours.

- Data saturation: the goal of the focus groups was to elicit a range of perspectives/insights from stakeholders for the initial intervention design. The focus groups were not designed to reach response saturation.

- Transcripts were not returned to participants for comment and/or correction.

**Domain 3: Analysis and findings**

***Data analysis***: Each transcript was reviewed for content analysis by 3 researchers. Some themes were identified in advance, based on the Focus Guide questions. A general inductive approach was used, where themes were also derived from the data. A formal coding tree is not applicable for the focus groups. Atlas.ti Qualitative Data Analysis software was employed to manage the data. Participants did not provide feedback on the findings.
***Reporting***: Quotations are presented in the manuscript text and Table S4. Quotations are identified by participant role (patient or caregiver). Areas of consistency (major themes) and a description diversity of responses (minor themes) are presented in the Results section of the manuscript, together with how the data impacted the intervention design.

**Pre-study Semi-structured Interview Questions**1) Why did you decide to participate in the *Being Present* study?

2) What are your expectations about practicing mindfulness meditation?

Do you think that mindfulness meditation will help? If so, how and why?

3) Do you have any prior experience with meditation, yoga or other mindfulness based

activities? 

4) Would you have been more or less likely to participate in this study if the mindfulness

trainings were offered as a smartphone app?

**Post-study Semi-structured Interview Questions**1) What was your experience with the *Being Present* study?

- what changes did you notice? [positive or negative]

- What did you like or find easy about the study?

- What aspects of the study were frustrating or difficult?

2) Overall, did you find the study to be helpful?

3) Where and when did you typically practice?

4) Did you continue practicing throughout the 8 weeks?

- if yes, what helped you to continue?

- if no, what got in the way of practice or what were your reasons for stopping?

5) Have you experienced any negative effects as a result of participating in this study?

6) Do you intend to practice mindfulness going forward? If so, how?

7) If you participated with a partner, what was that experience like?

8) Do you have any additional suggestions for how we could improve the study experience?

**Qualitative Data Analysis Plan for Semi-structured Interviews**

- 1. Comparison of different qualitative data analysis methods
  2. “Framework approach^2^”
     1. Deductive approach
     2. Summary: Keep the study objectives and interview questions in mind while looking for repeating themes and patterns in patient/caregiver interviews to learn about outcomes.
     3. *Categorical aggregation* as method to establish emergent themes.
  3. Steps summary
     1. Organize data: pre- and post-study patient/caregiver interviews were transcribed and labeled
     2. Familiarization with whole data set
        1. Reading, highlighting, taking notes, re-reading
     3. Create framework – develop **coding plan**: specific to broad
        1. Create **codes** – repeated themes and desired highlighted data, i.e., answers to questions asked in interviews; other codes arose from patient/caregiver frequent responses that were not asked specifically
        2. Codes defined by two investigators (M.C. and C.E.A)
     4. Indexing– code data by select quotes for **interpretation** of pre-determined codes.
        1. Atlas.ti Qualitative Data Analysis software employed
     5. Create **framework matrix** for analysis
        1. Independent review by M.C. and C.E.A
        2. Combine, diversify, and expand codes for final review
     6. Final review
         i. Data tabulation in a summary spreadsheet
         ii. Data analysis/interpretation (M.C. and C.E.A with input from A.A. and A.D.)

**Semi-structured Interviews COREQ**^1^

**Domain 1: Research team and reflexivity**

***Personal Characteristics*:** the interviewer was Blake Rosenthal, B.A., clinical research coordinator. Training was provided by G.J. and C.E. A. B.R. did not have a relationship with the participants prior to study commencement. Participants only knew of B.R. as the research coordinator.

**Domain 2: Study design**

***Theoretical framework****:* the methodological orientation was a framework approach.

***Participant selection***:
- Sampling: all *Being Present* intervention participants were asked to provide interviews.

- Method of approach: face-to-face or by telephone.

- Sample size: 45 pre-intervention interviews and 24 post-intervention interviews were conducted.

- Non-participation: the principal reasons for non-participation was worsening illness, including hospitalization or death and caregiving for a patient with worsening illness.

***Setting:***

- Setting of data collection: in clinic or by phone.

- Presence of non-participants: none.

- Description of sample: pre-intervention interviews were conducted with 28 patients and 17 caregivers; post-intervention interviews were conducted with 17 patients and 7 caregivers.

***Data collection:***

- The Interview Guides are provided in S1 File.

- Repeat interviews: pre- and post-intervention interviews were conducted wherever possible (N=24).

- Audio recordings were used to collect the data.

- Field notes were not made during or after the interviews.
- Duration: variable, roughly ten minutes per interview.

- Data saturation was reached for major themes.

- Transcripts were not returned to participants for comment and/or correction.

**Domain 3: Analysis and findings**

***Data analysis***:

- Number of data coders: two researchers (M.C. and C.E.A) coded the data.

- A description of the coding tree is provided in S1 File (see above).

- Themes were identified in advance, based on the interview questions, and also derived from the data.

- Software: Atlas.ti Qualitative Data Analysis software was used to manage the data.
 - Participants did not provide feedback on the findings.
***Reporting***: Quotations are presented in the manuscript text and Tables S6 and S7. Each quotation is identified by participant role (patient or caregiver), gender and age. There was consistency between the data presented and the findings. Major and minor themes are presented in Table 4 and 5 and are discussed in the manuscript.

**References**

1. Tong A, Sainsbury P, Craig J. Consolidated criteria for reporting

qualitative research (COREQ): a 32-item checklist for interviews and focus groups. International Journal for Quality in Health Care, 2001; 19: 349-357.

2. Gale NK, Heath G, Cameron E, Rashid, S, Redwood S. Using the framework method for the analysis of qualitative data in multi-disciplinary health research. BMC Medical Research Methodology. 2013;13:117.
