# Supplementary material for: Being Present: A single-arm feasibility study of audio-based mindfulness meditation for colorectal cancer patients and caregivers
Source: PLoS One. 2018 Jul 23;13(7):e0199423. doi: 10.1371/journal.pone.0199423 (PMC6056029; doi:10.1371/journal.pone.0199423)
Supplement: S2 File — With Institutional Review Board approval notification. (PDF) [file pone.0199423.s017.pdf]

UCSF Helen Diller Family Comprehensive Cancer Center

Being Present: Audio-Based Mindfulness Meditation Intervention for Colorectal Cancer Patients and Caregivers

Protocol Number: 15455
Study Drug: non-therapeutic

Version Number: 1.1
Version Date: 6/19/2015

IND Number: Exempt

Principal Investigator (Sponsor-Investigator)

Chloe E. Atreya, MD, PhD
550 16th St, 6th Floor
San Francisco, CA 94158
Telephone: 415-353-9888
E-mail: Chloe.Atreya@ucsf.edu

Co-Investigators

Alan P. Venook, MD
Anand A. Dhruva, MD
Andrew Avins, MD
Ai Kubo, PhD
Erin L. Van Blarigan, ScD
Dianne Shumay, PhD
Kara Bischoff, MD
Katherine Van Loon, MD
R. Kate Kelley, MD
Emily Bergsland, MD
Andrew Ko, MD
Galen Joseph, PhD
Hala Borno, MD

Biostatistician

Saunak Sen, PhD

Clinical Research Coordinator

Blake Rosenthal

Proprietary and Confidential

The information in this document is considered privileged and confidential, and may not be disclosed to others except to the extent necessary to obtain Institutional Review Board approval and informed consent, or as required by Federal and State laws. Persons to whom this information is disclosed should be informed that this information is privileged and confidential and that it should not be further disclosed.

Protocol Signature Page

Protocol No.: Pending **Version Date:** June 19th, 2015

1. I agree to follow this protocol version as approved by the UCSF Protocol Review Committee (PRC), Committee on Human Research (CHR), and Data Safety Monitoring Committee (DSMC).
2. I will conduct the study in accordance with applicable CHR requirements, Federal regulations, and state and local laws to maintain the protection of the rights and welfare of study participants.
3. I certify that I, and the study staff, have received the requisite training to conduct this research protocol.
4. I have read and understand the information in the Investigators' Brochure (or Manufacturer's Brochure) regarding the risks and potential benefits. I agree to conduct the protocol in accordance with Good Clinical Practices (ICH-GCP), the applicable ethical principles, the Statement of Investigator (Form FDA 1572), and with local regulatory requirements. In accordance with the FDA Modernization Act, I will ensure the registration of the trial on the www.clinicaltrials.gov website.
5. I agree to maintain adequate and accurate records in accordance with CHR policies, Federal, state and local laws and regulations.

UCSF Principal Investigator / Study Chair

Chloe E. Atreya, MD, PhD

Printed Name

Chloe Atreya June 19, 2015

Signature Date

Table of Contents

Protocol Signature Page 1
Abstract 3
1 Background 6
1.1 Rationale 7
1.2 Preliminary Data and Expertise 8
2 Study Aims 9
3 Study Design and Eligibility Criteria 10
3.1 Study Design 10
3.2 Inclusion Criteria 10
Eligibility Criteria 10
3.3 Exclusion Criteria 11
4 Patient Registration 11
5 Clinical Procedures, Data Collection, Intervention Details and Observations 11
5.1 Clinical Procedures and Data Collection 12
6 Schedule of Assessments 15
6.1 Screening Assessments 16
6.2 Clinical Assessments 16
6.3 Other Assessments 16
6.4 Criteria for Termination 16
7 Adverse Events 16
7.1 Adverse Event Reporting, Procedures, and Definitions 16
7.2 Follow-up of Adverse Events 17
7.3 Expedited Reporting 17
8 Statistical Considerations 17
8.1 Outcomes 17
8.2 Statistical Analyses 18
Table 8.2. Feasibility and Outcome Measures 18
8.3 Accrual Objectives 19
8.4 Estimated Duration of Study 19
8.5 Data Safety Monitoring 19
8.6 Analyses Time Points 20
8.7 Power and Sample Size Calculation 20
9 Ethical Aspects 20
9.1 Regulatory Considerations 20
9.2 Independent Ethics Committees/Institutional Review Board 20
10 Study Management 21
Pre-study Documentation 21
Institutional Review Board Approval 21
Informed Consent 21
Changes in the Protocol 21
11 Protection of Human Subjects 22
Protection of Privacy 22
Appendices 22
References 23

Abstract

Title	Being Present: Audio-Based Mindfulness Meditation Intervention for Colorectal Cancer Patients and Caregivers
Rationale for Study	A metastatic cancer diagnosis is associated with high levels of distress in both patients and caregivers. We hypothesize that an audio-based mindfulness intervention will be an effective means to reduce distress and improve quality of life among patients with advanced cancer and their caregivers. With the Being Present study, we will first establish patient and caregiver perceptions of audio-based mindfulness training via two focus groups among 8-10 patients with metastatic colorectal cancer and 8-10 caregivers. We will ask participants about intervention content, duration/frequency, and delivery; and perceived barriers/benefits to engaging in and adhering to mindfulness training. Focus group feedback will guide refinement of the intervention. We will then conduct an 8-week single arm study among UCSF patients with metastatic colorectal cancer undergoing chemotherapy and caregivers of these patients (44 participants total). Participants will receive an informational booklet containing a practice log and a MP3 player containing an introductory lecture and guided meditations. Practice reminders will be sent via text messages. Emails will contain practice assignments and links to validated survey instruments. The survey instruments (NCCN Distress Thermometer and NIH Patient Reported Outcomes Measurement Information System adult short forms) will measure global health, anxiety, depression, fatigue, and sleep quality before, during, and after the intervention to obtain preliminary estimates of the efficacy of the intervention among patients and caregivers on self-reported outcomes. Qualitative data will be collected from pre- and post-intervention interviews.
Study Design	Recruit and consent 8-10 patients with metastatic colorectal cancer and 8-10 caregivers for 2 focus groups<br>↓<br>Conduct two focus groups<br>↓<br>Refine intervention based on focus group feedback<br>↓<br>Recruit and consent patients and caregivers (44 total) for 8-week intervention study<br>↓<br>Conduct baseline assessments (demographics, medical history, DT, PROMIS, interview)<br>↓<br>Send text messages and weekly emails (practice reminders, instructions, and links to practice logs)<br>↓<br>Conduct mid- (week 4) & post-intervention (week 8) assessments (DT, PROMIS)<br>↓<br>Conduct exit Interview<br>↓<br>Send 3-month post-intervention follow-up email

Primary Aims	Establish patient and caregiver perceptions of audio-based mindfulness mediation training in order to refine the intervention for further study (Aim 1).<br><br>Assess feasibility and acceptability of the intervention among patients and caregivers (Aim 2).
Secondary Aims	Obtain preliminary estimates of efficacy of an audio-based mindfulness mediation intervention among patients and caregivers.
Number of patients	We propose to enroll 16-20 subjects in two focus groups (one with 8-10 HDFCCC patients with metastatic colorectal cancer and one with 8-10 caregivers of such patients). For the intervention study, we propose to enroll eligible patients and caregivers of these patients (n= 44 total).
Duration of Therapy	After baseline assessments, participants will listen to and practice with one track per day (20 minutes each), at least 5 days per week, for 8 weeks
Duration of Follow up	One follow-up email will be sent 3 months after study completion.
Duration of study	The total study duration is anticipated to equal approximately 6 months from first patient enrolled.
Intervention Device	Audio-based mindfulness tracks (MP3 format).

List of Abbreviations

AE	Adverse events
CTSI	Clinical and Translational Research Institute
DT	NCCN Distress Thermometer
HADS	Hospital Anxiety and Depression Scale
HDFCCC	Helen Diller Family Comprehensive Cancer Center
MBSR	Mindfulness-Based Stress Reduction
NCCN	National Comprehensive Cancer Network
OCIM	Osher Center for Integrative Medicine
PROMIS	NIH Patient Reported Outcomes Measurement Information System
QOL	Quality of life
RCT	Randomized controlled trial
REDCap	Research Electronic Data Capture system
SMS	Symptom Management Service

1 Background

The National Comprehensive Cancer Network (NCCN) definition of distress is "a multifactorial unpleasant emotional experience of a psychological (cognitive, behavioral, emotional), social, and/or spiritual nature that may interfere with the ability to cope effectively with cancer, its physical symptoms and its treatment. Distress extends along a continuum, ranging from common normal feelings of vulnerability, sadness, and fears to problems that can become disabling, such as depression, anxiety, panic, social isolation, and existential and spiritual crisis."[1] An estimated 35% of cancer patients experience psychological distress.[2] Research indicates that addressing distress may improve survival in addition to quality of life (QOL).[3] Screening for psychosocial distress will become a Cancer Center accreditation requirement in 2015.

To help cope with distress associated with the diagnosis, disease symptoms, and treatment, cancer patients are increasingly turning to complementary and alternative medicine,[4-8] including mindfulness practices and meditation. Mindfulness can be defined as "paying attention on purpose, in the present moment, and nonjudgmentally, to the unfolding experience moment-by-moment".[9] By repeatedly bringing attention back to the immediate experience, practitioners gradually learn to disengage from reactivity and dwelling on the past or the future; instead directly experiencing current emotions and bodily sensations. Mindfulness practice, commonly offered in US medical settings as Mindfulness-Based Stress Reduction (MBSR)[9] programs or Mindfulness-Based Cognitive Therapy,[10] has shown efficacy among cancer patients in reducing psychological distress and improving quality of life (QOL).[11] MBSR has also been associated with decreased levels of cortisol and pro-inflammatory cytokines; lower blood pressure and modified brain activity.[12-15]

Despite evidence of effectiveness of mindfulness-based programs,[11] the majority of previous oncology studies were conducted among early stage breast cancer patients who had already completed treatment. Ongoing chemotherapy typically indicates more advanced stage cancer, which causes substantial distress for both patients and caregivers. Due to disease symptoms and/or treatment side-effects such as fatigue, anorexia and diarrhea, such patients and their caregivers are rarely good candidates for attending weekly in-person classes (MBSR includes 31 hours of in-person instruction). Additionally, because UCSF is a referral center, patients often travel from great distances for treatment or a second opinion, again diminishing the practicality of an in-person intervention. For these reasons, the proposed research will evaluate the feasibility and acceptability of delivering an audio-based mindfulness intervention to oncology patients with metastatic disease who are receiving chemotherapy.

Caregivers (a partner, other family member, or close friend) will be included in the Being Present study. Caregivers experience high levels of anxiety, depression, fatigue and sleep disturbance,[16-21] yet family caregivers of cancer patients have been subjects of only two prior mindfulness studies. Birnie et al found that MBSR improved mood and

stress levels in both cancer patients (mostly early stage) and their partners (n=21 couples).[22] The investigators observed that partners' moods correlated with patients' stress level. Post-intervention scores in patient-partner pairs were more aligned than at baseline. It was suggested that participating in MBSR as a couple might improve adherence as well as responses to relationship stress. In the only study of patients with advanced stage cancer and their caregivers, Lengacher et al assessed a modified-MBSR program (n=26 dyads), including 3 in-person classes over 6 weeks.[12] The investigators observed improved psychological scores in both patients and caregivers, with greater benefit among patients, and a high rate of retention. Of note however, over 50% of screened patients who declined participation cited "schedule conflict", "lives too far", "caregiver unavailable", or "no transportation" as the reason.

The focus population for our audio-based intervention will be patients with metastatic colorectal cancer. Colorectal cancer is the second leading cause of cancer death in the US, accounting for an estimated 50,000 deaths in 2014.[23] Approximately half of patients with colorectal cancer present with or develop distant metastases (stage IV), typically representing incurable disease. Although patients with metastatic colorectal cancer continue to have a poor prognosis, today they are living longer than ever before. In a recent US phase III trial, led by Alan Venook, MD, comparing two standard of care chemotherapy regimens for metastatic colorectal cancer, and observed a median overall survival of 29 months.[24] During this time, anxiety about the future and other cancer-associated symptoms are major sources of distress.[25-27] To date, no mindfulness studies have focused on colorectal cancer patients. An obvious difference from breast cancer is that about half of colorectal cancer patients are male. Focusing on metastatic colorectal cancer will enable us to compare recruitment and adherence in male and female patients, together with their partners or other caregivers. Other significant differences from breast cancer include the treatment regimens and the presence of an ostomy in some patients. Patients with metastatic small bowel adenocarcinomas will also be eligible, as these rare intestinal neoplasms are treated like colorectal cancer.

1.1 Rationale

Limitations of available mindfulness training options (in particular, dependence on in-person classes) severely restrict their potential to benefit those most in need: patients with metastatic cancer receiving chemotherapy and their caregivers. In the Being Present study, we will evaluate the feasibility, acceptability and efficacy of an audio-based mindfulness intervention in UCSF Helen Diller Family Comprehensive Cancer Center (HDFCCC) patients with metastatic colorectal cancer and their caregivers. The inclusion of caregivers is an important component of this pilot. Few prior mindfulness studies have included caregivers. We hypothesize that the patient-caregiver dyad will be especially important for the success of an intervention without group classes. The patient-caregiver relationship may serve as a source of mutual support and a surrogate for community, which is traditionally considered to be an essential ingredient for

sustaining mindfulness practices. Although this type of intervention may be useful to patients with other cancer types, we have chosen to focus on metastatic colorectal cancer because it is understudied, highly prevalent, and also because patients with metastatic colorectal cancer tend to receive similar treatments and live for many months with relatively stable health, enabling study completion. We posit that an intervention that complements chemotherapy to help manage the psychological, social, and physical challenges associated with metastatic colorectal cancer may improve the QOL of both patients and their caregivers.

Positive preliminary data from this pilot study would provide support for a large scale randomized controlled trial (RCT) to evaluate the novel delivery of this safe, simple, and inexpensive mindfulness intervention (which will be developed into a mobile application) to reduce distress and improve QOL among cancer patients undergoing chemotherapy and their caregivers. Positive results from the larger RCT would yield an effective tool for clinicians to offer to distressed cancer patients and all those who care about them.

1.2 Preliminary Data and Expertise

A feasibility pilot study using an audio CD-based mindfulness intervention for patients receiving chemotherapy was conducted by our co-investigator, Dr. Avins, who holds joint appointments in the Departments of Medicine and Epidemiology & Biostatistics at UCSF and the Division of Research at Kaiser Permanente Northern California and is an OCIM affiliated faculty member.[28] Patients listened to and practiced mindfulness exercises similar to those taught in didactic, in-person MBSR classes. In this initial pilot study, any cancer patient undergoing chemotherapy was eligible to enroll: no effort was made to recruit a diverse study population. The study included mostly breast cancer patients (n=13), although there were small numbers of patients with a variety of other cancers. Only 2 of 23 participants had CRC; 21 of 23 patients were female; 20 of 23 patients completed the study protocol. Mean Hospital Anxiety and Depression Scale (HADS) scores decreased by 6.1 points (95% CI, −2.9 to −9.4). Caregivers were not evaluated. The study demonstrated the feasibility of this intervention from the perspective of both patients and clinicians, and provided preliminary evidence of its efficacy in improving depression and QOL[28], establishing it as a rational basis for this proposal.

Dr. Dhruva, a medical oncologist and integrative medicine physician at HDFCCC and OCIM, conducts research on non-pharmacologic integrative medicine interventions for cancer symptom management and supportive care. He studies the biology and clinical trajectory of cancer associated symptoms in both oncology patients and their family caregivers.[29-34]. In his research, he has consistently observed comparable levels of fatigue, depression and other symptoms in cancer patients and family caregivers, supporting inclusion of caregivers in this proposal. Dr. Dhruva has also conducted a pilot study of yoga breathing for cancer chemotherapy-associated symptoms and QOL.[35] In his clinical practice, he has extensive experience evaluating and treating symptoms, including fatigue and depressive symptoms. Dr. Kubo is a cancer

epidemiologist in the UCSF Clinical and Translational Research Institute (CTSI) K Scholar Program. She is also a certified yoga instructor with a depth of knowledge in mindfulness and yoga. Dr. Kubo conducted a pilot yoga intervention study for racially/ethnically diverse heart failure patients to improve QOL and depression[36]. The Principal Investigator of this study, Dr. Atreya, is a medical oncologist at HDFCCC whose clinical practice focuses on metastatic colorectal cancer. Under the mentorship of Dr. Venook, Dr. Atreya is experienced at conducting clinical trials and translational research studies in this patient population at UCSF.[37-39] As a CTSI K Scholar, Dr. Atreya earned Advanced Training in Clinical Research Certification. Pertinent to the Being Present study, she completed MBSR training at the OCIM in 2013.

2 Study Aims

Aim 1: Establish patient and caregiver perceptions of audio-based mindfulness meditation training in order to refine the intervention for further study.

We will conduct a qualitative study and obtain data via two focus groups among 1) 8-10 HDFCCC patients with metastatic colon, rectum, or small bowel adenocarcinoma and 2) 8-10 caregivers of such patients. We will ask participants about intervention content, duration/frequency, and delivery, as well as perceived barriers/benefits to engaging in and adhering to mindfulness meditation training. Focus group feedback will be used to improve the intervention.

Aim 2: Test the feasibility, acceptability, and initial estimates of efficacy of an audio-based mindfulness meditation intervention among patients and caregivers.

We will conduct an 8-week single arm pilot study among HDFCCC patients with metastatic colorectal cancer receiving chemotherapy and their caregivers (44 participants, total). Participants will receive an informational booklet containing a practice log and an MP3 player containing an introductory lecture and guided meditations. Practice reminders will be sent via text messages. Weekly emails will contain practice instructions and links to validated questionnaires.

- We will assess the feasibility and acceptability of the intervention by evaluating the recruitment rate, reasons for non-participation/non-continuation of the intervention, and adherence (via practice logs and responses to text messages). Interviews with participants will provide qualitative data about the intervention experience.
- We will obtain preliminary estimates of the efficacy of the intervention on self-reported outcomes using validated questionnaires to measure global health, anxiety, depression, fatigue, sleep quality, and mindfulness before, during, and after the intervention. We hypothesize that the audio-based mindfulness

meditation intervention will reduce distress in both patients with metastatic colorectal cancer and their caregivers.

3 Study Design and Eligibility Criteria

3.1 Study Design

Aim 1 will be focus groups and Aim 2 will be an uncontrolled pilot intervention study.

3.2 Inclusion Criteria

We propose to enroll 16-20 subjects in two focus groups (one with 8-10 HDFCCC patients with metastatic colorectal cancer and one with 8-10 caregivers of such patients). For the intervention study, we propose to enroll eligible patients and caregivers of these patients (n= 44 total).

The main purpose of the focus groups is to inform the design of the intervention for the pilot study, therefore we will use similar inclusion/exclusion criteria for both aims. All participants must be adults who are able to provide informed consent.

Eligibility Criteria

Eligible patients must:

- Carry a diagnosis of metastatic colon, rectum, or small bowel adenocarcinoma
- Anticipate receiving chemotherapy for at least 12 weeks total from the time of recruitment (ongoing chemotherapy not required for focus group participants)
- Have life expectancy of at least 6 months
- Have Karnofsky Performance Status ≥60
- Be able to speak and read English
- Have access to a mobile phone
- Be able to navigate websites, fill out forms on the web, communicate by email, and have regular access to the internet
- Have a distress level of ≥3 on the NCCN Distress Thermometer (DT).[1, 40] This is required for intervention group participants only to be able to show improvement in mood/QOL.
- An effort will always be made to recruit patient and caregiver pairs, but unpaired patients are also eligible

Eligible caregivers are a spouse/partner, other family member, or a close friend of a patient with metastatic colon, rectum, or small bowel adenocarcinoma.
Eligible caregivers must:

- Be able to speak and read English
- Have access to a mobile phone
- Be able to navigate websites, fill out forms on the web, communicate by email, and have regular access to the internet
- Unpaired caregivers, including former caregivers of patients who are now

deceased, are eligible for the focus group only.

3.3 Exclusion Criteria

We are limiting the study to patients with metastatic colon, rectum, or small bowel adenocarcinoma, because treatment regimens and disease trajectories for these disease groups are similar.

Deafness, current meditation practice (>2 episodes or >1 hour total, weekly), and current enrollment in a stress reduction program are exclusion criteria for the intervention study because the study is designed as an audio-based introduction to mindfulness meditation. Patients with a DT level >7 will be considered on a case-by-case basis: in consultation with Drs. Shumay and Bischoff, efforts will be made to address sources of high distress prior to study enrollment. Patients and caregivers who participate in a focus group and meet criteria for the intervention will be given the opportunity to participate: data will be collected, but these patients/participants will not be included in the final analyses so as not to bias the results.

4 Patient Registration

This will be an uncontrolled pilot intervention study. All patients and caregivers who are consented will be entered into the UCSF Cancer Center OnCore database. The OnCore database is password protected and meets HIPAA guidelines. All patient information contained in OnCore is strictly confidential and is treated as Protected Health Information, as defined in 45 CFR 164.501. Data entered into OnCore is stored behind university firewalls in a secure Oracle database located at the UCSF Mission Bay Data Coordinating Center, which is maintained by UCSF Translational Informatics Staff. REST based web services over SSL will convey the data to the UCSF Research Electronic Data Capture system (REDCap) where familiar forms and statistical package export facilities can be utilized by researchers to track study progress, export data and perform analyses.

5 Clinical Procedures, Data Collection, Intervention Details and Observations

A written, signed, informed consent form (ICF) and a Health Insurance Portability and Accountability Act (HIPAA) authorization must be obtained before any study-specific assessments are initiated. A copy of the signed ICF will be given to the subject and a copy will be filed in the medical record. The original will be kept on file with the study records.

5.1 Clinical Procedures and Data Collection

AIM 1: Conduct two focus groups to assess perceptions of audio-based mindfulness meditation

Recruitment Methods: Medical records of patients will be reviewed by the study team to assess initial eligibility. Potentially eligible participants will primarily be recruited from the GI Oncology, OCIM, and SMS Clinics. Candidates seen in clinic will be given an informational flyer about the study and will be asked for permission to be contacted by the research coordinator. A secondary recruitment mechanism, for study candidates without an upcoming clinic appointment, will be an invitation letter. The invitation letter will tell candidates about the study and inform them that a member of our research team will call them about the study in 1-2 weeks, unless they do not want to be called (a stamped postcard will be included for patients to send back to indicate that they do not want to be called). The research coordinator will contact candidates who express interest in clinic or who do not opt-out by mail to further describe the study using a semi-structured script. If the individuals are still interested, the clinical research coordinator will go through a series of questions to determine final eligibility and coordinate scheduling. We will initially invite 12 individuals per group (patients and caregivers), and increase the number invited as needed based on number of refusals, to achieve 8-10 participants. We will limit participation to the first 10 respondents in each category. To reduce participant burden, we will provide a meal, parking, and a $30 gift card.

Session Format: We will conduct two focus groups among 1) 8-10 HDFCCC patients with metastatic colon, rectum, or small bowel adenocarcinoma and 2) 8-10 caregivers of such patients to assess perceptions of audio-based mindfulness meditation training, with a goal to improve the feasibility, acceptability, and efficacy of the intervention. Focus groups will be designed and conducted by Dr. Galen Joseph, Associate Professor in the Department of Anthropology, History, and Social Medicine. Dr. Joseph has experience conducting focus groups among breast and colon cancer survivors. We will also consult with Dr. Shumay, Associate Director of Psycho-Oncology at HDFCCC, and cancer advocates at UCSF to ensure that the materials are appropriate for our population of interest. Treating providers will not attend the focus groups to facilitate open discussion.

Table 5.1. Focus Group Agenda

Focus Group Agenda
**I. Introduction; warm up questions**
• Assess knowledge about mindfulness practices: working definitions, types (e.g. meditation, yoga), experience
• Perceived barriers & benefits to engaging in mindfulness practices at this time in one's life
**II. Demonstrate intervention; gather feedback**
• Delivery: feasibility of using MP3 player, texts & email; goal to subsequently create a mobile app
• Audio files: acceptability to patients & caregivers of content, including voices; duration; schedule
• Texts and emails: content and schedule, for purpose of increasing adherence & monitoring efficacy
• Pros and cons of offering intervention to patient & caregiver pairs; no in-person sessions

Intervention Refinement: Following focus group feedback, MP3 files of modified audio scripts will be generated under the direction of co-investigator, Dr. Kubo. From our previous experience, it will take one month to finalize the audio files, together with improvements to the text and email content

AIM 2: Conduct an 8-week pilot study of audio-based mindfulness meditation intervention

Recruitment Methods*:*

Clinicians will recruit eligible patients and caregivers of these patients (n= 44 total) from the HDFCCC GI Oncology and OCIM, and SMS Clinics. Medical records of candidate patients will be reviewed by the study team to assess initial eligibility. Candidates seen in clinic will be given an informational flyer about the study and will be asked for permission to be contacted by the research coordinator. Recruitment from GI Oncology Clinic is an important component of this proposal as we seek to offer mindfulness training to patients who do not access OCIM due to lesser preexisting interest in complementary therapies or practical considerations such as distance traveled, treatment schedule, and physical limitations. In 2013, 135 new patients with metastatic colorectal cancer were seen in the GI Oncology Clinic. Patients seen in follow-up who meet the eligibility criteria are also candidates. Candidates who do not have an upcoming appointment at UCSF may be sent an invitation letter that tells them that we will call them in 1-2 weeks to discuss the study. The letter will also include a stamped postcard for them to return to indicate that they do not want to be called.

***Initial Research Coordinator Contact*:** A clinical research coordinator will contact potentially interested study candidates. Key details of the study, such as the rationale and the time commitment required will be explained using a semi-structured script. If a candidate remains interested in participating, the clinical research coordinator will go through a series of questions to determine final eligibility and coordinate scheduling of an in-person meeting (consent visit), typically on the day of a chemotherapy infusion. If an eligible participant is unable to come into clinic, the research coordinator will send the participant a secure e-mail link to an online consent form via Redcap. After consent is obtained, the research coordinator will mail the MP3 player and booklet and set up a study-initiation call. Candidates determined to be ineligible, including interested caregivers of patients who decline participation, will be provided with a list of resources for psychosocial support (e.g. contact information for Psycho-Oncology and Social Work) as well as resources for mindfulness practice (e.g. MBSR courses at OCIM).

***Study Initiation*:** A research coordinator or study investigator will obtain informed consent after a candidate demonstrates comprehension of the consent form and has been given ample opportunity to ask questions. Each participant will receive an MP3 player (theirs to keep) with recorded instructions and mindfulness exercises as well as a booklet with written instructions and a practice log. Study staff will explain how to use the exercises and to keep track of meditation practice (practice frequency and duration;

tracks listened to). A brief interview will assess expectations prior to starting the intervention. Demographic information (e.g. age, gender, race/ethnicity, education, socioeconomic status, marital/partner status, and sexual orientation) and medical history (e.g. chemotherapy regimen, treatment history; other pertinent medications and comorbidities) will be collected from the participant and/or electronic medical record. Baseline assessments will be conducted using validated questionnaires on an encrypted laptop or tablet, with responses collected in REDCap. We will use instruments that are brief (combined time ~12 minutes to complete): DT and the NIH Patient Reported Outcomes Measurement Information System (PROMIS) [41] short forms for global health, anxiety, depression, fatigue and sleep disturbance as well as the Five Facet Mindfulness Questionnaire (FFMQ) [42] short form.

Audio-based Mindfulness Meditation Exercises: These exercises (full transcripts in Appendix), developed by MBSR teachers, are designed to relax the physical body and improve mindfulness. Introduction of mindfulness exercises will be staggered as below to allow participants to progressively familiarize themselves with each modality and gain competence to choose exercises according preference and circumstances, increasing self-reliance. Each meditation track lasts about 20 minutes. Participants will listen to and practice with one track per day, at least 5 days per week, for 8 weeks (the same duration as the MBSR program).

Table 5.2. Audio-based Mindfulness Tracks

5.2	Week	MP3 Tracks to guide practice
**5.3**	**1**	*5.4* 1. Introduction;<br>*5.5* 2. Mindful Breathing
**5.6**	**2**	5.7 3. Progressive Muscle Relaxation
**5.8**	**3**	5.9 4. Safe Place Meditation
**5.10**	**4**	5.11 5. Attention Awareness Meditation
**5.12**	**5**	5.13 6. Body Awareness Meditation
**5.14**	**6**	5.15 7. Loving Kindness Meditation

**5.16**	**7**	*Any tracks of your choice*
**5.17**	**8**	8. Self-Guided Meditation

Emails and Text Messages: Weekly emails will contain practice assignments and a theme for the week (again, similar to the MBSR curriculum), tips, and/or links to survey assessments (data collected on REDCap). Text messages will serve as practice reminders. Participants will receive text messages containing motivational quotes, tips, and queries requesting a 1-2 character response (Y, N or a number). We will develop text message content and timing during focus group sessions. The research team will have access to password-protected, real-time population and individual patient dashboards throughout the study. At the end of the study, the text message service will transfer all archived message data, including sender, recipient, time stamp, and content, to the research team.

Mid- and Post-intervention Assessments **(weeks 4 and 8):** During the 4th week of the intervention, an email with a unique link to online survey instruments (DT, PROMIS, and FFMQ) will be sent. A reminder will automatically be resent to participants who do not complete the survey questions within 5 days. Participants may also opt to complete the survey on an encrypted computer during a clinic or infusion visit. If no response after the reminder email, the research coordinator will call the participant and conduct a phone assessment using the same instruments or send a paper copy. The process will be repeated at week 8 (see timetable). A brief follow-up assessment will be sent via email 3 months after study completion.

Exit interview**:** After the 8-week intervention period, study staff (excluding the treating physician) will conduct an in-person or phone interview to collect qualitative data regarding participants' experience of the intervention. For example, we will ask about the effects of the intervention; what was easy or difficult; what aspects will be continued or not and why; reasons for stopping (for those who decided not to continue); and input on how we can improve the exercises. The research coordinator will obtain a copy of the practice log at the exit interview.

Ineligible/refusal log**:** For potential participants who are ineligible or who express interest in the study but then decide not to participate, we will log demographic and medical information, caregiver relationship, and reasons for ineligibility/refusal as feasibility/acceptability measures to guide design of subsequent studies.

6 Schedule of Assessments

Table 6. Study Calendar

Study Timetable	Week 0	1	2	3	4	5	6	7	8
Eligibility screening	X								
Informed consent		X							
Demographics and medical history		X							
Qualitative assessments: interview questions		X							X
Participant-reported outcomes: DT, HADS, PROMIS		X			X				X
Practice reminders (text messages)		X	X	X	X	X	X	X	X
Practice logs (online, link in email)		X	X	X	X	X	X	X	X

6.1 Screening Assessments

HDFCCC patients with metastatic colon rectum, or small bowel adenocarcinoma, and caregivers of such patients, who meet the eligibility criteria (Section 3.0) will be introduced to the study. Our screening procedure is described in detail in Section 3.3.

6.2 Clinical Assessments

There will be no in-person clinical assessments in this study.

6.3 Other Assessments

Questionnaire assessments and timing are described in Section 5.2. All instruments will be reviewed/approved by IRB prior to administration.

6.4 Criteria for Termination

The Principal Investigator may terminate the participation of an individual patient if they withdraw informed consent. If a participant withdraws, we will ask if s/he would still be willing to complete the surveys and an exit interview.

7 Adverse Events

7.1 Adverse Event Reporting, Procedures, and Definitions

We are testing an audio-based meditation program as the intervention, and all potential participants will be screened via the process outlined in Section 3.3 to assess contraindications to participation. There are no anticipated *a priori* adverse events (AE's) for this intervention. Study participants will be encouraged to report any 'emergencies or events' by calling the CRC or PI. These instructions will be included in the consent form and study booklet. Additionally, participants will be asked in their exit interview if they experienced any adverse events as a result of participating in the Being Present study? The PI will record all reported events in an adverse event log (including subject's name, date, and event description – see attached log entry form). Dr. Atreya will consult with the co-investigators on the action to be taken. This action and date of

implementation will also be recorded in the adverse event log. The entire investigative team will participate in classifying events as 'serious' or 'non-serious' (see listed below), as well as 'non-attributable', 'possibly attributable', or 'attributable' to the proposed study.

Adverse Event Definitions

Serious – any event or condition that is life threatening, results in a hospitalization, or cancer or a physical or cardiac event serious enough to require medical attention. A brief listing follows:

- Death
- Life-threatening (places the subject, in the view of the investigator, at immediate risk of death from the reaction as it occurred),
- Inpatient hospitalization or prolongs existing hospitalization,
- Persistent or significant disability/incapacity (a substantial disruption of a person's ability to conduct normal life functions),
- Birth defect/congenital anomaly,
- Or any important medical event that may not result in prior listed outcomes but, based upon appropriate medical judgment, may jeopardize the subject, and may require medical or surgical intervention to prevent one of the prior listed outcomes.

Non-Serious – all other events

7.2 Follow-up of Adverse Events

All adverse events will be followed with appropriate medical management until resolved. Patients removed from study for unacceptable adverse events will be followed until resolution or stabilization of the adverse event. For selected adverse events for which administration of the mindfulness intervention was stopped, a re-challenge of the subject with the mindfulness intervention may be conducted if considered both safe and ethical by the Investigator.

7.3 Expedited Reporting

Reporting to UCSF Committee on Human Research (Institutional Review Board)

The Principal Investigator must report events meeting the UCSF CHR definition of "Unanticipated Problem" (UP) within 10 business days of his/her awareness of the event.

8 Statistical Considerations

8.1 Outcomes

- **Primary Outcomes**

Feasibility and acceptability of audio-based mindfulness meditation training: We will assess feasibility and acceptability of the intervention among patients and caregivers by evaluating the recruitment rate, reasons for non-participation/non-continuation of the intervention, and adherence (via practice logs and responses to text messages). Interviews with participants will provide qualitative data about the intervention experience

- **Secondary Outcomes**

Efficacy of audio-based mindfulness meditation training: We will obtain preliminary estimates of the efficacy of the intervention among patients and caregivers on self-reported outcomes using validated questionnaires to measure global health, anxiety, depression, fatigue, sleep quality, and mindfulness before, during, and after the intervention. We hypothesize that the audio-based mindfulness meditation intervention will reduce distress in both patients with metastatic colon, rectum, or small bowel cancer and their caregivers.

8.2 Statistical Analyses

We will use descriptive statistics for the feasibility and outcome measures, including the proportion of eligible patients and caregivers who agree to be screened; consent to participate; and complete or partially complete the intervention. Reasons for ineligibility; refusal to participate in or complete the intervention will be tabulated. Logistic regression models will assess demographic and clinical characteristics associated with consent to participate among eligible and screened patients and caregivers. Qualitative thematic analysis will characterize interview data. Effect sizes will be calculated by comparing baseline and end of study participant-reported outcomes related to QOL, distress, anxiety, depression, fatigue, sleep quality, and mindfulness. We will also conduct repeated measures analyses (e.g. mixed effects linear models with a random intercept for participant) that include the mid-point time-point data. Efficacy analyses will be exploratory in nature. Where applicable, measures of feasibility, acceptability and efficacy will be compared in patient-caregiver dyads and between paired and unpaired participants.

Table 8.2. Feasibility and Outcome Measures

Data Collected Measures:	Feasibility	Acceptability	Efficacy
Focus group feedback	X	X	
Recruitment rate	X	X	
Reasons for ineligibility	X		
Reasons for refusal to participate	X	X	
Study completion rate	X		
Adherence	X		
Demographics & medical history	X	X	
Qualitative assessments (interviews)	X	X	X
Participant-reported outcomes (surveys)			X

8.3 Accrual Objectives

Our target enrollment is 16-20 subjects for the focus groups (8-10 patients with metastatic colon, rectum, or small bowel cancer and 8-10 caregivers of such patients) and 44 participants in the intervention study. Focus group participants who also participate in the intervention will not count toward the accrual goal for the intervention (n=44), as they will be excluded from the final analysis. To meet the accrual numbers, we estimate researchers will screen/approach ~100 potential participants (patients and caregivers). Based on our recruitment strategy, we estimate enrollment duration of five months (1 month for the focus groups; 4 months for the intervention study).

8.4 Estimated Duration of Study

This study is funded for one year.

Table 8.4. Funding Calendar

Month of Funding	1	2	3	4	5	6	7	8	9	10	11	12
Obtain CHR approval	X	X										
Development of survey platform & text messaging	X	X										
Recruit and conduct focus groups, refine intervention content			X	X	X							
Recruitment for pilot intervention study					X	X	X	X				
Conduct 8-week pilot intervention study					X	X	X	X	X	X		
Data analysis, abstract/manuscript and grant-writing											X	X

8.5 Data Safety Monitoring

Trial Type/Level of Review: Pilot Behavioral Trial – This study does not involve the testing of pharmacologic agents or any therapeutic treatments. It is a non-randomized behavioral trial designed to examine feasibility, acceptance, and behavior change for patients provided with audio-based training in mindfulness meditation. Thus, it is classified as a Type 2 Study (Non-Therapeutic Intervention Study), a minimal risk level study that dictates annual review by the Cancer Center Scientific Monitoring Subcommittee for scientific progress and IRB compliance at UCSF. We do not anticipate the need for a Data Safety Monitoring Board (DSMB); however we will take

this under advisement from our protocol review committees. Ultimately, the PI will be responsible for the conduct of this study and will review the AE procedures monthly (see Section 10).

8.6 Analyses Time Points

Statistical analyses for all aims will be conducted in January and February 2016.

8.7 Power and Sample Size Calculation

There are no statistical analyses for **Aim 1**; therefore we did not conduct a power or sample size calculation. The proposed sample size of 8-10 participants per focus group was chosen based on the expertise of Dr. Galen Joseph[43]. For intervention feasibility (**Aim 2**), our power calculation is based on the proportion of participants who successfully complete the intervention, defined as ≥70% of participants listening to the meditation recordings and completing the 8-week follow-up assessment. Using a 1-sided 1-sample binomial test with $\alpha = 0.05$, 20 patients or caregivers, and an expected successful completion proportion of 70%, we will have 80% power to reject the null hypothesis if 48% or fewer participants successfully complete the protocol. In other words, the intervention will be considered *not* feasible for patients if fewer than 10 patients complete the protocol, and *not* feasible for caregivers if fewer than 10 caregivers complete the protocol.

9 Ethical Aspects

There are few risks associated with participation and potentially gains in education regarding the health benefits of mindfulness meditation. The PI will confirm that the basic principles of informed consent are met: autonomy, beneficence, justice, and non-maleficence.

9.1 Regulatory Considerations

The UCSF GI Oncology Site Committee, the UCSF Comprehensive Cancer Center Protocol Review Committee, and the UCSF Committee on Human Research (IRB; see below) will review this study.

9.2 Independent Ethics Committees/Institutional Review Board

The UCSF Committee on Human Research (IRB) approval for this protocol and the informed consent will be sought before the study moves forward. The PI is responsible for keeping the IRB advised of the progress of the study and of any changes made in the protocol prior to implementation. The PI will also keep the IRB informed of any significant adverse reactions, and any protocol exceptions or deviations. Records of all study review and approval documents must be kept on file by the PI and are subject to inspection during or after completion of the study. The IRB will receive notification of the termination of the study.

10 Study Management

Pre-study Documentation

This study will be conducted in accordance with the ethical principles that have their origin in the Declaration of Helsinki as stated in 21 CFR §312.120(c)(4); consistent with GCP and all applicable regulatory requirements.

Before initiating this trial, the Investigator will have written and dated approval from the Institutional Review Board for the protocol, written informed consent form, subject recruitment materials, and any other written information to be provided to subjects before any protocol related procedures are performed on any subjects.

The clinical investigation will not begin until either FDA has determined that the study under the Investigational Drug Application (IND) is allowed to proceed or the Investigator has received a letter from FDA stating that the study is exempt from IND requirements.

Institutional Review Board Approval

The protocol, the proposed informed consent form, and all forms of participant information related to the study (e.g. advertisements used to recruit participants) will be reviewed and approved by the UCSF CHR (UCSF Institutional Review Board). Prior to obtaining CHR approval, the protocol must be approved by the Helen Diller Family Comprehensive Cancer Center Site Committee and by the Protocol Review Committee (PRC). The initial protocol and all protocol amendments must be approved by the IRB prior to implementation.

Informed Consent

All participants must be provided a consent form describing the study with sufficient information for each participant to make an informed decision regarding their participation. Participants must sign the CHR-approved informed consent form prior to participation in any study specific procedure. The participant must receive a copy of the signed and dated consent document. The original signed copy of the consent document must be retained in the medical record or research file.

Changes in the Protocol

Once the protocol has been approved by the UCSF CHR, any changes to the protocol must be documented in the form of an amendment. The amendment must be signed by the Investigator and approved by PRC and the CHR prior to implementation.

If it becomes necessary to alter the protocol to eliminate an immediate hazard to patients, an amendment may be implemented prior to CHR approval. In this

circumstance, however, the Investigator must then notify the CHR in writing within five (5) working days after implementation. The Study Chair and the UCSF study team will be responsible for updating any participating sites.

11 Protection of Human Subjects

Protection of Privacy

Patients will be informed of the extent to which their confidential health information generated from this study may be used for research purposes. Following this discussion, they will be asked to sign the HIPAA form and informed consent documents. The original signed document will become part of the patient's medical records, and each patient will receive a copy of the signed document. The use and disclosure of protected health information will be limited to the individuals described in the informed consent document.

Appendices

A1.	Informed consent forms for focus groups	
A2.	Informed consent form for intervention	
A3.	Flyer	*revised 6.19.15*
A4.	Invitation letter for focus groups	
A5.	Invitation letter for intervention	
A6.	Semi-structured script introducing focus groups	
A7.	Semi-structured script introducing intervention	
A8.	Focus group guide	
A9.	Demographic survey	*revised 6.19.15*
A10.	Surveys (DT, PROMIS, FFMQ)	*revised 6.19.15*
A11.	Transcript of mindfulness exercises	*revised 6.19.15*
A12.	Weekly emails	
A13.	Weekly themes	
A14.	Resources for further practice	
A15.	Study booklet	
A16.	Text messaging grid	

References

1. National Comprehensive Cancer Network. Distress Management Clinical Practice Guidelines in Oncology. Version 1.2014.

2. Zabora J, Brintzenhofe, Szoc K, Curbow B et al. The prevalence of psychological distress by cancer site. Psychooncology 2001; 10: 19-28.
3. Giese-Davis J, Collie K, Rancourt KM et al. Decrease in depression symptoms is associated with longer survival in patients with metastatic breast cancer: a secondary analysis. J Clin Oncol 2011; 29: 413-420.
4. Greenlee H, Kwan ML, Ergas IJ et al. Complementary and alternative therapy use before and after breast cancer diagnosis: the Pathways Study. Breast cancer research and treatment 2009; 117: 653-665.
5. Shumay DM, Maskarinec G, Gotay CC et al. Determinants of the degree of complementary and alternative medicine use among patients with cancer. Journal of alternative and complementary medicine 2002; 8: 661-671.
6. Yates JS, Mustian KM, Morrow GR et al. Prevalence of complementary and alternative medicine use in cancer patients during treatment. Supportive care in cancer : official journal of the Multinational Association of Supportive Care in Cancer 2005; 13: 806-811.
7. Huebner J, Prott FJ, Micke O et al. Online survey of cancer patients on complementary and alternative medicine. Oncology research and treatment 2014; 37: 304-308.
8. Huebner J, Muenstedt K, Prott FJ et al. Online survey of patients with breast cancer on complementary and alternative medicine. Breast care 2014; 9: 60-63.
9. Kabat-Zinn J. Full catastrophe living : using the wisdom of your body and mind to face stress, pain, and illness New York, New York Delta Trade Paperbacks 1990.
10. Segal ZV, Williams JMG, Teasdale JD. Mindfulness based cognitive therapy for depression: A new approach to preventing relapse. New York, NY: Guilford Press 2002.
11. Piet J, Wurtzen H, Zachariae R. The effect of mindfulness-based therapy on symptoms of anxiety and depression in adult cancer patients and survivors: a systematic review and meta-analysis. J Consult Clin Psychol 2012; 80: 1007-1020.
12. Lengacher CA, Kip KE, Barta M et al. A pilot study evaluating the effect of mindfulness-based stress reduction on psychological status, physical status, salivary cortisol, and interleukin-6 among advanced-stage cancer patients and their caregivers. J Holist Nurs 2012; 30: 170-185.
13. Carlson LE, Speca M, Faris P, Patel KD. One year pre-post intervention follow-up of psychological, immune, endocrine and blood pressure outcomes of mindfulness-based stress reduction (MBSR) in breast and prostate cancer outpatients. Brain Behav Immun 2007; 21: 1038-1049.
14. Bhasin MK, Dusek JA, Chang BH et al. Relaxation response induces temporal transcriptome changes in energy metabolism, insulin secretion and inflammatory pathways. PLoS One 2013; 8: e62817.

15. Holzel BK, Hoge EA, Greve DN et al. Neural mechanisms of symptom improvements in generalized anxiety disorder following mindfulness training. Neuroimage Clin 2013; 2: 448-458.
16. Golant M, Haskins NV. "Other cancer survivors": the impact on family and caregivers. Cancer J 2008; 14: 420-424.
17. Hodges LJ, Humphris GM, Macfarlane G. A meta-analytic investigation of the relationship between the psychological distress of cancer patients and their carers. Soc Sci Med 2005; 60: 1-12.
18. Segrin C, Badger TA, Harrington J. Interdependent psychological quality of life in dyads adjusting to prostate cancer. Health Psychol 2012; 31: 70-79.
19. Given CW, Stommel M, Given B et al. The influence of cancer patients' symptoms and functional states on patients' depression and family caregivers' reaction and depression. Health Psychol 1993; 12: 277-285.
20. Carney S, Koetters T, Cho M et al. Differences in sleep disturbance parameters between oncology outpatients and their family caregivers. J Clin Oncol 2011; 29: 1001-1006.
21. Fletcher BS, Paul SM, Dodd MJ et al. Prevalence, severity, and impact of symptoms on female family caregivers of patients at the initiation of radiation therapy for prostate cancer. J Clin Oncol 2008; 26: 599-605.
22. Birnie DH, Sears SF, Green MS et al. No long-term psychological morbidity living with an implantable cardioverter defibrillator under advisory: the Medtronic Marquis experience. Europace 2009; 11: 26-30.
23. Benson AB, 3rd, Venook AP, Bekaii-Saab T et al. Colon cancer, version 3.2014. J Natl Compr Canc Netw 2014; 12: 1028-1059.
24. Venook A, Niedzwiecki D, Lenz H-J et al. CALGB/SWOG 80405: Phase III trial of irinotecan/5-FU/leucovorin (FOLFIRI) or oxaliplatin/5-FU/leucovorin (mFOLFOX6) with bevacizumab (BV) or cetuximab (CET) for patients (pts) with KRAS wild-type (wt) untreated metastatic adenocarcinoma of the colon or rectum (MCRC). J Clin Oncol 32:5s, 2014 (suppl; abstr LBA3).
25. Artherholt SB, Fann JR. Psychosocial care in cancer. Curr Psychiatry Rep 2012; 14: 23-29.
26. Fann JR, Ell K, Sharpe M. Integrating psychosocial care into cancer services. J Clin Oncol 2012; 30: 1178-1186.
27. Smets EM, Garssen B, Schuster-Uitterhoeve AL, de Haes JC. Fatigue in cancer patients. Br J Cancer 1993; 68: 220-224.
28. Altschuler A, Rosenbaum E, Gordon P et al. Audio recordings of mindfulness-based stress reduction training to improve cancer patients' mood and quality of life--a pilot feasibility study. Support Care Cancer 2012; 20: 1291-1297.
29. Dhruva A, Aouizerat BE, Cooper B et al. Cytokine Gene Associations With Self-Report Ratings of Morning and Evening Fatigue in Oncology Patients and Their Family Caregivers. Biol Res Nurs 2014.
30. Dhruva A, Aouizerat BE, Cooper B et al. Differences in morning and evening fatigue in oncology patients and their family caregivers. Eur J Oncol Nurs 2013; 17: 841-848.

31. Merriman JD, Aouizerat BE, Langford DJ et al. Preliminary evidence of an association between an interleukin 6 promoter polymorphism and self-reported attentional function in oncology patients and their family caregivers. Biol Res Nurs 2014; 16: 152-159.
32. Dunn LB, Aouizerat BE, Langford DJ et al. Cytokine gene variation is associated with depressive symptom trajectories in oncology patients and family caregivers. Eur J Oncol Nurs 2013; 17: 346-353.
33. Miaskowski C, Cooper BA, Dhruva A et al. Evidence of associations between cytokine genes and subjective reports of sleep disturbance in oncology patients and their family caregivers. PLoS One 2012; 7: e40560.
34. Dhruva A, Lee K, Paul SM et al. Sleep-wake circadian activity rhythms and fatigue in family caregivers of oncology patients. Cancer Nurs 2012; 35: 70-81.
35. Dhruva A, Miaskowski C, Abrams D et al. Yoga breathing for cancer chemotherapy-associated symptoms and quality of life: results of a pilot randomized controlled trial. J Altern Complement Med 2012; 18: 473-479.
36. Kubo A, Hung YY, Ritterman J. Yoga for heart failure patients: a feasibility pilot study with a multiethnic population. Int J Yoga Therap 2011; 77-83.
37. Bendell J, Atreya C, André T et al. Efficacy and tolerability in an open-label phase I/II study of MEK inhibitor trametinib (T), BRAF inhibitor dabrafenib (D), and anti-EGFR antibody panitumumab (P) in combination in patients (pts) with BRAF V600E mutated colorectal cancer (CRC). J Clin Oncol 32:5s, 2014 (suppl; abstr 3515).
38. Warren RS, Atreya CE, Niedzwiecki D et al. Association of TP53 mutational status and gender with survival after adjuvant treatment for stage III colon cancer: results of CALGB 89803. Clin Cancer Res 2013; 19: 5777-5787.
39. Atreya CE, Sangale Z, Xu N et al. PTEN expression is consistent in colorectal cancer primaries and metastases and associates with patient survival. Cancer Med 2013; 2: 496-506.
40. Carlson LE, Waller A, Mitchell AJ. Screening for distress and unmet needs in patients with cancer: review and recommendations. J Clin Oncol 2012; 30: 1160-1177.
41. Broderick J, DeWitt E, Rothrock N et al. Advances in Patient-Reported Outcomes: The NIH PROMIS® Measures. eGEMs 2013; 1: Article12.
42. Baer RA, Smith GT, Hopkins J et al. Using self-report assessment methods to explore facets of mindfulness. Assessment 2006; 13: 27-45.
43. Berg B. Qualitative Research Methods for the Social Sciences 6th ed. Pearson Education Inc. 2006

UCSF
University of California
San Francisco

Human Research Protection Program
Committee on Human Research

Notification of Expedited Review Approval

Principal Investigator
Chloe E Atreya

Co-Principal Investigator

Type of Submission: Modification Form
Study Title: CC# 15455: Being Present: Audio-Based Mindfulness Meditation Intervention for Colorectal Cancer Patients and Caregivers

IRB #: 15-16158
Reference #: 141703

Committee of Record: Mount Zion Panel

Study Risk Assignment: Minimal

Approval Date: 07/07/2015 **Expiration Date:** 04/12/2018

Regulatory Determinations Pertaining to this Approval (if applicable):

The proposed changes constitute: Minor

IRB Comments (if applicable):

All changes to a study must receive CHR approval before they are implemented. Follow the modification request instructions. The only exception to the requirement for prior CHR review and approval is when the changes are necessary to eliminate apparent immediate hazards to the subject (45 CFR 46.103.b.4, 21 CFR 56.108.a). In such cases, report the actions taken by following these instructions.

Expiration Notice: The iRIS system will generate an email notification eight weeks prior to the expiration of this study's approval. However, it is your responsibility to ensure that an application for continuing review approval has been submitted by the required time. In addition, you are required to submit a study closeout report at the completion of the project.

Approved Documents: To obtain a list of documents that were approved with this submission, follow these steps: Go to My Studies and open the study – Click on Submissions History – Go to Completed Submissions – Locate this submission and click on the Details button to view a list of submitted documents and their outcomes.

For a list of all currently approved documents, follow these steps: Go to My Studies and open the study – Click on Informed Consent to obtain a list of approved consent documents and Other Study Documents for a list of other approved documents.

San Francisco Veterans Affairs Medical Center (SFVAMC): If the SFVAMC is engaged in this research, you must secure approval of the VA Research & Development Committee in addition to CHR approval and follow all applicable VA and other federal requirements. The CHR website has more information.
